# Characterization of the Phenolic Fingerprint of *Kolovi* Extra Virgin Olive Oils from Lesvos with Regard to Altitude and Farming System Analyzed by UHPLC-QTOF-MS

**DOI:** 10.3390/molecules26185634

**Published:** 2021-09-17

**Authors:** Natasa P. Kalogiouri, Evangelia Kritikou, Ioannis C. Martakos, Constantina Lazarou, Michalis Pentogennis, Nikolaos S. Thomaidis

**Affiliations:** Laboratory of Analytical Chemistry, Department of Chemistry, National and Kapodistrian University of Athens, Panepistimiopolis Zographou, 15771 Athens, Greece; kalogiourin@chem.uoa.gr (N.P.K.); evkritik@chem.uoa.gr (E.K.); johnmrtk@chem.uoa.gr (I.C.M.); clazarou@chem.uoa.gr (C.L.); pentogmi@otenet.gr (M.P.)

**Keywords:** olive oil, *Kolovi*, secoiridoids, QTOF-MS, phenolic content, health claim

## Abstract

Extra virgin olive oil (EVOO) is recognized for its nutritional virtues and the beneficial health effects deriving from its hydrophilic fraction (phenolic acids, phenolic alcohols, flavonoids, and secoiridoids). The phenolic compounds of EVOOs possess multiple biological properties such as antioxidant, antimicrobial, anticarcinogenic, and anti-inflammatory properties, among others. Considering that EVOOs produced in Greece are recognized as high-quality products due to their rich phenolic content, it is imperative to characterize Greek monovarietal EVOOs and ensure that their uniqueness is closely linked to their botanical and territorial origin. In this work, an ultra-high-performance liquid chromatography–quadrupole time-of-flight tandem mass spectrometry (UHPLC-QTOF-MS) analytical method combined with target and suspect screening was used to characterize monovarietal EVOOs of the *Kolovi* variety from Lesvos, and thereby establish their phenolic fingerprint. Overall, 25 phenols were determined, and the total quantification and semi-quantification results ranged between 251 and 1230 mg/kg, highlighting the high phenolic content of the *Kolovi* variety from the island of Lesvos in the North Aegean.

## 1. Introduction

Olive oil, the emblematic food of the Mediterranean diet, is recognized for its nutritional value and health benefits. Olive oil holds a powerful and special place at the base of the Mediterranean Diet Pyramid as the principal source of fat, consisting of low concentrations of saturated fat and high concentrations of monounsaturated and polyunsaturated fat, as well as a variety of other bioactive compounds [1,2]. Furthermore, numerous studies have associated olive oil consumption with lower mortality rates of cardiovascular disease and cancer indices, as well as eliminated risk for neurodegenerative diseases, among others, and better well-being [3,4,5,6,7,8].

The nutritional value, health properties, flavor, and taste have been associated with olive oil’s minor constituents of the polar phenolic fraction, phenolic compounds. However, the phenolic profile varies among cultivars and critically depends on the geographical origin [9,10,11], the type of farming [12,13], and the production system [14,15], among other factors, such as irrigation, fertilization practices, and pruning, in combination with several technological factors [16].

The olive tree *Olea europaea* L. has diverged naturally into many cultivars in the Mediterranean basin. A total of 69% of global olive oil production takes place in Spain, Italy, Greece, Portugal, France, Slovenia, Croatia, Cyprus, and Malta, while Spain, Italy, Greece, and Portugal together account for about 99% of the production in the E.U. [17]. As far as Greece is concerned, even though the total number of the olive cultivars is greater than 40, more than 90% of the territory is cultivated with 20 of them (*Agouromanakolia*, *Adramytiani*, *Amigdalolia*, *Athinolia*, *Asprolia*, *Valanolia*, *Vasilikada*, *Gaidurelia*, *Dafnelia*, *Thiaki*, *Kalamon*, *Kalokerida, Karolia*, *Karidolia*, *Kothreiki*, *Kolompada*, *Konservolia*, *Koroneiki*, *Koutsourelia*, *Lianolia*, *Kerkiras*, *Kalamon*, *Kalokerida*, *Karolia*, *Karidolia*, *Kothreiki*, *Kolimpada*, *Konservolia*, *Koroneiki*, *Koutsourelia*, *Lianolia*, *Kerkiras*, *Mavrelia*, *Megaritiki*, *Mittolia*, *Strogilolia*, *Throumbolia*, *Tragolia*) [18]. Greece holds a prominent place in the olive oil and table olives market. According to the International Olive Council (IOC), Greece occupies the first place in the consumption of olive oil per capita per year (16 kg), while at the same time it is ranked as the world’s leading exporter of Extra Virgin Olive Oil (EVOO) [19].

One of the largest cultivation territories of Greece is the island of Lesvos. EVOOs originating from Lesvos are allowed to market under Protected Geographical Indication (PGI) [EU No 1151/2012] [20]. In Lesvos, traditional Greek varieties such as *Koroneiki* and *Ladoelia* are cultivated alongside local varieties of *Kolovi*, *Adramytiani*, and *Agrielia*. Recent studies have revealed the high nutritional value of olive oils produced by olive fruits of the *Kolovi* variety. Even though the fingerprints of other Greek cultivars belonging to different varieties, such as *Koroneiki* [21,22], *Kalamon* [23] etc., have already been described, limited data are available about the *Kolovi* variety. A few reports are available concerning the determination of bioactive constituents, such as phenolic compounds, tocopherols, carotenoids, and squalene [24,25,26]. Still, there are no studies available presenting an in-depth study of the phenolic fraction of olive oils produced by *Kolovi* olive fruits.

Several works have been published for the determination of phenolic constituents in EVOOs with traditional analytical methodologies employing High Pressure Liquid Chromatography (HPLC) coupled to UV [27,28,29] or Diode Array Detection (DAD) [30,31,32]. Gas Chromatography (GC) [33,34] is not widely applied due to the low volatility of phenolic compounds. Nuclear Magnetic Resonance (NMR) methodologies enable the determination of a wide range of phenolic compounds [35,36,37]. The revolution of “omics” technologies, however, has introduced high-throughput analytical techniques in food profiling studies through target and non-target analysis. High-Resolution Mass Spectrometry (HRMS) enables the detection of a great number of features, following the “foodomics approaches” that can be grouped as “profiling”, analyzing target analytes, or “fingerprinting”, which is based on the analysis of the whole food metabolome [38]. Target screening is based on the determination of already known analytes with commercially available standards, and suspect screening is applied for the analysis of compounds that are expected to exist in the matrix, and can be screened using the exact mass of their molecular ions, and follow a specific flow chart with diagnostic criteria for their tentative identification [38,39,40]. In this respect, HRMS enables the analysis of analytes for which there are no available commercial standards, and thus they cannot be determined by traditional analytical methodologies. The use of ultra-high-performance liquid chromatography coupled to quadrupole time-of-flight mass spectrometry (UHPLC-QTOF-MS) enables the tentative identification of suspect phenolic compounds in EVOOs providing high mass accuracy, making possible to generate isotopic patterns, improving the accuracy of chemical formula prediction, and library matching. Considering that there are no available commercial analytical standards for several phenolic phytochemicals found in EVOOs, the latest analytical challenge is the determination of the EVOOs polar fraction, raised after the establishment of the health claim for EVOOs that contain 5 mg of hydroxytyrosol and derivatives (e.g., oleuropein complex and tyrosol) per 20 g oil, according to the EC Reg. 432/2012 [41]. Additionally, the bioactive profile is closely associated with the olive cultivar [42] and the geographical origin [43], along with the farming system (organic or conventional) [26], and several other processing factors [36,37], and thus it is worth evaluating the impact of such agronomical parameters on the total phenolic content of monovarietal EVOOs.

The purpose of this study was to investigate and highlight the phenolic profile of monovarietal EVOOs of the *Kolovi* variety by target and suspect UHPLC-QTOF-MS. Moreover, an investigation is carried out regarding the concentration levels of phenolic compounds and their changes with altitude, and the type of farming (organic or conventional) in monovarietal EVOOs produced by two-phase centrifugation systems, within a two-year study.

## 2. Results and Discussion

### 2.1. Phenolic Profiling and Fingerprinting Results

#### 2.1.1. Target Screening Results

A target data-dependent method was employed to scan the presence of 14 target compounds (caffeic acid, ferulic acid, gallic acid, homovanillic acid, p-coumaric acid, syringic acid, hydroxytyrosol, tyrosol, vanillin, apigenin, epicatechin, luteolin, oleuropein, and pinoresinol) in real *Kolovi* EVOO samples. Five compounds were determined. These were tyrosol and hydroxytyrosol from the class of phenolic alcohols, apigenin and luteolin from the class of flavonoids, and pinoresinol from the class of lignans. The retention time (t_R_) shift was less than 0.04 min for all the detected compounds. The mass accuracy of the precursor and qualifier ions was lower than 2.5 mDa, compared to the standard solutions, and the isotopic fit was lower than 100 mSigma in all cases. The fragments were verified on the basis of MS/MS records in the literature [24,26]. The target screening results are presented in Table 1.

For the quantification of the target phenolic compounds, normalized calibration curves were constructed for hydroxytyrosol, tyrosol, apigenin, luteolin, and pinoresinol. Mixed standard working solutions were prepared before analysis by appropriate dilution of the stock solutions with methanol:water (80:20, *v*/*v*) in the concentration range 0.1–12 mg/L. For each concentration level (0.1, 1, 2, 4, 8 and 12 mg/L), the peak area of each standard was divided by the peak area of the internal standard (syringaldehyde 1.3 mg/L). Indicative normalized standard calibration curves of tyrosol, hydroxytyrosol, oleuropein, apigenin, luteolin and pinoresinol for one laboratory day, with r^2^ above 0.99, are presented in Table S1, in the Supplementary Information. The identified phenolic compounds were further quantified, and the target quantification results, expressed as mg/kg, are presented in Table S2.

Hydroxytyrosol and tyrosol are the main phenolic alcohols identified in olive oils, and were determined in the ranges 0.15–59.0 mg/kg and 0.36–46.5 mg/kg, respectively. Hydroxytyrosol exhibits antioxidant, anti-inflammatory, and chemopreventive properties, while tyrosol has antioxidant and anti-inflammatory action [44]. Furthermore, hydroxytyrosol has been indicated to have a radio-protective effect on human skin [45]. In addition, hydroxytyrosol has been shown to improve tumoral and cardiac diseases with effects similar to those of oleuropein, while it protects against atherosclerosis and against diabetic neuropathy, as well [46].

From the class of flavonoids, apigenin was determined in the range 0.14–13.9 mg/kg, and luteolin in the range 0.22–7.71 mg/kg. Both flavonoids exhibit strong antioxidant activity, protecting against cancer [47]. From the class of lignans, pinoresinol was determined in the range 0.45–11.5 mg/kg, and has been shown to exhibit strong antioxidant and anti-inflammatory properties [48].

#### 2.1.2. Suspect Screening Results

A suspect list with 95 phenolic compounds, including all the possible secoiridoid derivatives of the oleuropein complex, previously published from our group [26], was used to scan their potential occurrence in the analyzed EVOOs. The initial suspect list is presented in Table S3. Overall, 20 phenolic compounds were tentatively identified in *Kolovi* EVOOs through suspect screening. The t_R_ shift of the compounds did not exceed ±0.18 min, and was compared to the corresponding predicted t_R_ of an in-house Quantitative Structure-Retention Relationship (QSRR), already published by our group [23]. Hydroxytyrosol acetate, decarboxymethyl lingstroside aglycone (oleocanthal), decarboxymethyl oleuropein aglycone (oleacein), 10-hydroxy-10-methyl oleuropein aglycone, 10-hydroxy-decarboxymethyl oleuropein aglycone, 10-hydroxy oleuropein aglycone, lingstroside aglycone, lingstroside aglycone monoaldehydic form, lingstroside aglycone dialdehydic form, oleokoronal, methyl oleuropein aglycone, oleuropein aglycone, oleuropein aglycone monoaldehydic form, oleuropein aglycone dialdehydic form, oleomissional, 1-acetoxypinoresinol, 1-hydroxypinoresinol, syringaresinol, elenolic acid and the hydroxylated form of elenolic acid were tentatively identified after examining the MS/MS spectra with Metfrag [49] and literature records [23,26,50]. Table 2 presents the suspect screening results, providing information about the MS/MS fragmentation of each compound, and the experimental t_R_.

The Extracted Ion Chromatograms (EICs) of lingstroside aglycone (Figure S1) and oleuropein aglycone (Figure S2) showed four different peaks. Lingstroside and oleuropein are stabilized by the presence of the lingstroside residue. The removal of the glucose exposes the labile hemiacetal carbon that undergoes ring opening resulting in a series of subsequent transformations [51,52].

**Figure 1:**
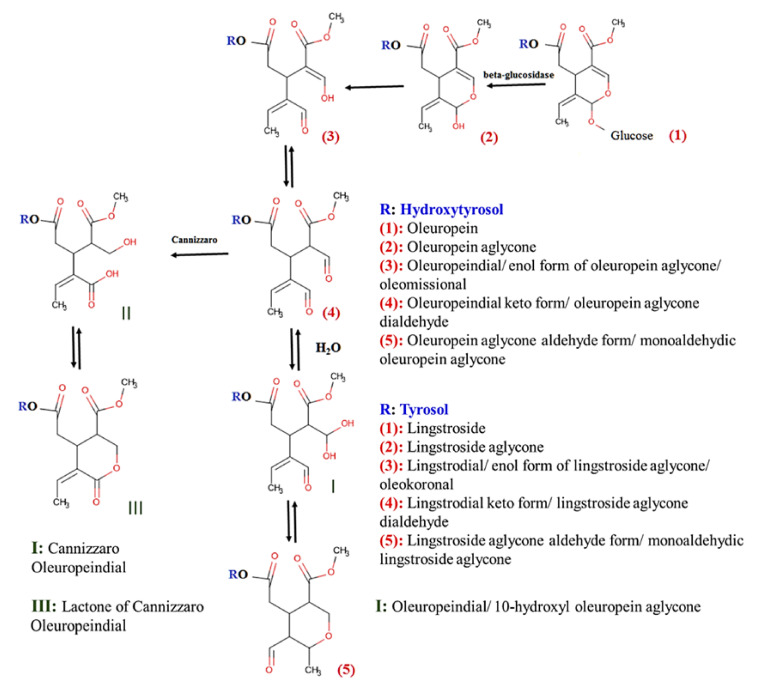
Biotransformation pathway of oleuropein and lingstroside.

Figure 1 presents a proposed series of reactions of oleuropein and lingstroside. Compound I (10-hydroxy oleuropein aglycone) was identified in all the analyzed samples. Compound (4) (oleuropein aglycone dialdehydic form) after reaction with the extracting solvent (methanol:water) transforms to 10-hydroxy-10-methyl oleuropein aglycone, which after dehydration converts to methyl oleuropein aglycone. Oleuropein aglycone, the enol form of oleuropein aglycone (also referred as oleuropendial or oleomissional [53]), the dialdehyde of oleuropein aglycone, and monoaldehyhydic oleuropein aglycone were tentatively identified, according to Kalogiouri et al. [26]. The MS/MS spectra with the characteristic *m*/*z*_s_ explained are presented in Figure S3. The derivatives of lingstroside, i.e., lingstroside aglycone, the enol form of lingstroside aglycone (also referred as oleokoronal [53]), the dialdehyde of lingstroside aglycone, and the monoaldehydic lingstroside aglycone were tentatively identified, as well, as shown in Table 2. The compounds II and III—the cannizzaro product and its lactone, shown in Figure 1—were not identified in any of the analyzed EVOOs.

The suspect compounds were semi-quantified on the basis of the commercially available standards for each class. In this respect, the lignans 1-acetoxypinoresinol, 1-hydroxypinoresinol and syringaresinol were semi-quantified with the calibration curve of pinoresinol; hydroxytyrosol acetate, oleocanthal, oleacein, 10-hydroxy decarboxymethyl oleuropein aglycone, elenolic acid and the hydroxylated form of elenolic acid were quantified with the calibration curve of hydroxytyrosol; 10-hydoxy-10-methyl oleuropein aglycone, 10-hydroxy oleuropein aglycone, the four isomers of lingstroside aglycone, methyl oleuropein aglycone and the four isomers of oleuropein aglycone were quantified with the calibration curve of oleuropein. The semi-quantification of all the individual phenolic compounds determined in the analyzed EVOOs are presented in Table S4, expressed as mg/kg.

Oleuropein is the major secoiridoid in olives. It decreases during olive oil processing to form derivatives. The sum of oleuropein aglycone, oleuropein aglycone monoaldehydic form, oleuropein aglycone dialdehydic form, and oleomissional ranged between 6.49 and 732 mg/kg. This dominant phenolic substance of VOOs/EVOOs is gaining attention due to its valuable biological properties, such as antioxidative, anti-inflammatory, anti-breast-cancer, anti-Alzheimer’s-disease, anti-hyperglycemic-effect, and lipid-lowering properties. The second most abundant secoiridoid was lingstroside aglycone, ranging between 8.21 and 360 mg/kg. Lingstroside aglycone exhibits antioxidant activity and anticarcinogenic properties [5], and it was recently referred to in the literature as a novel nutraceutical against osteoarthritis [6]. Decarboxymethyl oleuropein aglycone (oleacein), which has been shown to exhibit hypotensive, antimicrobial and anti-inflammatory properties [7], offering skin protection and reduction of disorder due to metabolic syndrome [54], was determined in the range 0.89–55.1 mg/kg. Moreover, decarboxymethyl lingstroside aglycone (oleocanthal), which has attracted great interest after a recent study that reported its strong anti-inflammatory capacity, acting similarly to the non-steroidal anti-inflammatory drug ibuprofen [8], was determined in the range 3.27–31.9 mg/kg. High concentrations of oleuropein derivatives were determined, as well. The highest determined concentration of methyl oleuropein aglycone was 732 mg/kg, followed by 10-hydroxy-10-methyl oleuropein aglycone (up to 99.2 mg/kg), followed by 10-hydroxy-oleuropein aglycone (up to 33.3 mg/kg), and 10-hydroxy-decarboxymethyl-oleuropein aglycone (up to 5.04 mg/kg). Hydroxytyrosol acetate, a hydroxytyrosol derivative, ranged between 2.30 and 28.9 mg/kg. The highest concentration observed for elenolic acid was 7.16 mg/kg, while this was 0.57 mg/kg for hydroxylated elenolic acid. From the class of lignans, 1-hydroxypinoresinol ranged between 0.19–2.87 mg/kg, while higher concentrations were determined for acetoxypinoresinol and syringaresinol, 84.4 mg/kg and 289 mg/kg, respectively.

The identified secoiridoids demonstrate favorable health effects in relation to oxidative stress and antithrombotic profiles, cardiovascular risk factors, blood pressure and lipids profile, endothial dysfunction, postprandial hyperlipidemia, acting against chronic diseases such as cancer, obesity, and diabetes [55].

**Figure 2:**
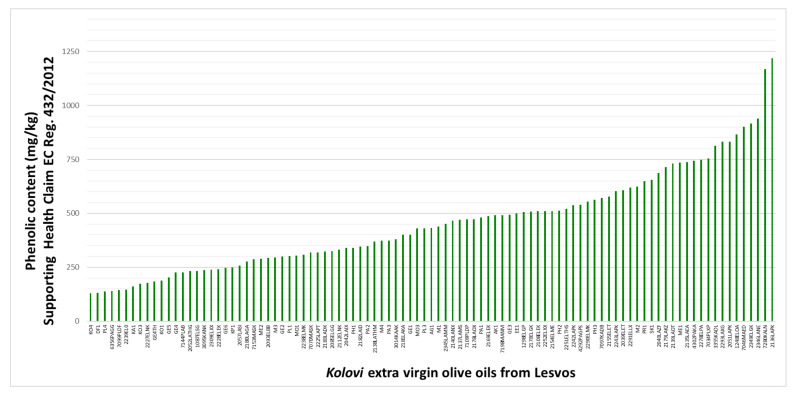
Phenolic content (mg/kg) of *Kolovi* EVOOs from Lesvos.

Even though the scientific issue concerning which compounds should be included in EC Reg. 432/2012 [41] is still under discussion, and no specific method has yet been adopted in the regulation for measuring olive oil polyphenols, Tsimidou et al. [56] recently proposed the quantification of tyrosol and hydroxytyrosol, in an attempt to clarify which compounds should be summed up to give the amount of free or in bound forms, with some groups supporting that individual phenolics should be summed up to give the amount of 250 mg per 1 kg of olive oil. According to this, tyrosol, hydroxytyrosol and their derivatives were summed up, and 76 out of the total 91 monovarietal *Kolovi* EVOOs, corresponding to 78% of the analyzed samples, were found to support the Health Claim, as graphically illustrated in Figure 2. The phenolic content supporting EC Reg. 432/2012 ranged between 130 and 1218 mg/kg, and 78% of the analyzed *Kolovi* EVOOs could be labeled as “contributing to the protection of blood lipids from oxidative stress”.

### 2.2. Phenolic Content and Agronomical Factors

Several factors interfere with the synthesis of phenolic compounds in EVOOs. Among them, the olive cultivar [42], the geographic origin [43], the climate [57], the degree of maturation [58], the crop season [59], postharvest storage, crushing, and malaxation, as well as after-production storage, affect the phenolic composition [36,37].

It is generally accepted that in monovarietal EVOOs of the same origin, the farming type and the altitude play a significant role. Even though the characterization of a large number of VOOs/EVOOs with reference to only one factor at a time is not an easy task, great effort was made to collect EVOOs harvested during the same period (between December and January) within a two-year study to detect any potential changes in the climate conditions. The collected samples were processed under similar conditions, i.e., produced with two-phase centrifugation systems, malaxation temperature up to 30 °C, and stored in glass bottles to facilitate the comparisons of their phenolic contents in terms of altitude and farming type (organic or conventional)**.**

#### 2.2.1. Altitude

The comparison of the quantification results of all the target analytes and the semi-quantification results of all the suspects in comparison with the altitude showed that the phenolic content was higher in EVOOs originating from medium and high altitudes for the two harvesting periods. Specifically, the average phenolic content of EVOOs originating from low altitudes (below 100 m) was equal to 361 mg/kg during the harvesting period 2016–2017, and 375 mg/kg during the harvesting period 2017–2018, respectively. The phenolic content of EVOOs originating from territories with medium (100–300 m) and high altitude (300–600 m), was higher compared to those originating from lower altitude, 454 mg/kg and 442 mg/kg during the harvesting period 2016–2017, respectively. The same applied for the phenolic content of the analyzed samples of the following harvesting period (2017–2018), with an average phenolic content of 562 mg/kg for those originating from territories with medium altitude, and 563 mg/kg for those originating from territories with high altitude. These results are in accordance with a recent work by Theodosi et al. [60], stating that the quality characteristics of olive oils change depending on the altitude, reporting that the total phenolic content augments with altitude level [60]. Obviously, there is a positive correlation between the climatic and geographic parameters and the production zones in the phenolic composition of the EVOOs. In contrast, another work by Mousa et al. [61] showed that the phenolic content was higher in EVOOs originating from territories lower than 100 m, compared to samples grown at 800 m. In this case, the disagreement with our results is perhaps due to the large differences in temperature in such high-altitude ranges, or the local pedoclimatic conditions, and rainfall, suggesting that altitude does not have a definitive influence, and may be counteracted by the effects of other geoclimatic factors, as well [43]. In agreement with Borges et al. [43] and Dabbou et al. [62], positive correlation was found between the concentration of polyphenols and altitude.

**Figure 3:**
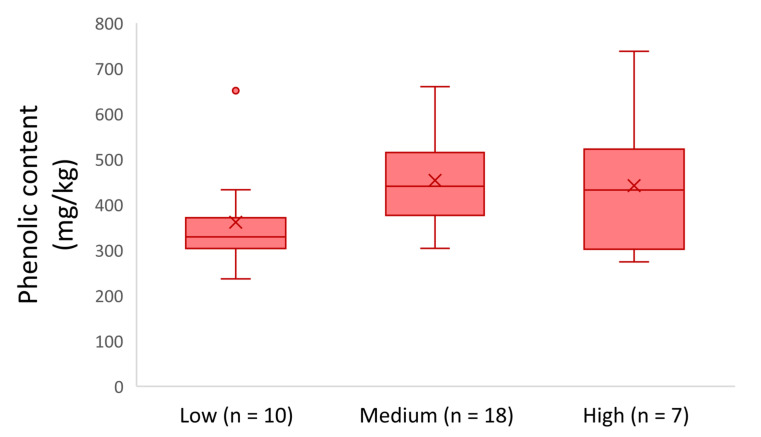
Phenolic content of the analyzed EVOOs produced in 2016–2017 with respect to the altitude.

Figure 3 shows that the EVOOs produced during the harvesting period 2016–2017 in territories with low altitude presented a lower phenolic content on average (361 mg/kg). Moreover, the olive oils from medium and high altitude had a higher phenolic content on average (454 mg/kg and 442 mg/kg, respectively). Analysis of Variance (ANOVA) showed that there was a statistically significant difference between the EVOOs from territories with low and medium altitude of cultivation (p = 0.038). In other cases, the differences were not statistically significant (*p*-value < 0.05).

**Figure 4:**
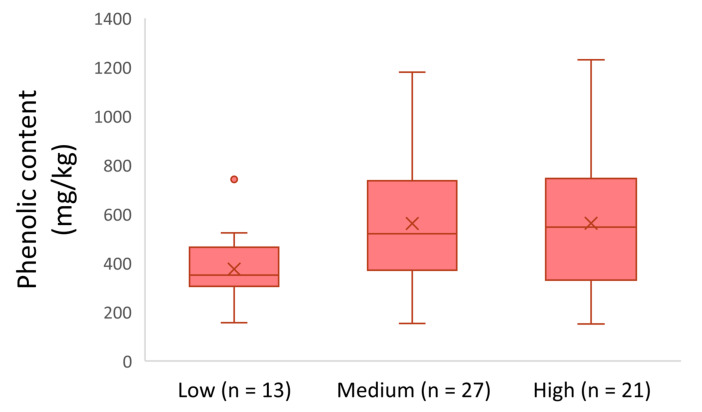
Phenolic content of the analyzed EVOOs produced in 2017–2018 with respect to altitude.

Figure 4 shows that the EVOOs produced during the harvesting period 2017–2018, originating from territories with low altitude presented a lower phenolic content on average (375 mg/kg). Moreover, the olive oils from medium and high altitude had a higher phenolic content on average (562 mg/kg and 563 mg/kg, respectively). ANOVA showed that the olive olives from locations with medium and high altitude of cultivation did not differ statistically (*p* = 0.99). On the other hand, the olive oils from locations at low altitude had a statistically significant difference compared to the olive oils from locations at medium and high altitude (*p*-value < 0.05).

Τhe average concentrations of the major target phenolic compounds, tyrosol and hydroxytyrosol, were also statistically analyzed to evaluate the concentration of the individual compounds was affected by altitude. During the crop year 2016–2017, the average concentration of hydroxytyrosol was equal to 17.2 mg/kg in EVOOs from low-altitude territories, 13.6 mg/kg in those originating from medium-altitude territories, and 22.0 mg/kg in EVOOs originating from cultivars at higher altitude. ANOVA showed that the concentration of hydroxytyrosol did not differ statistically between the analyzed EVOOs (*p* = 0.418). The average concentration of hydroxytyrosol during the crop year 2017–2018 was 2.29 mg/kg in EVOOs originating from low altitude, 3.00 mg/kg in EVOOs from medium-altitude cultivars, and 4.36 mg/kg in EVOOs originating from territories at high altitude, respectively. ANOVA showed that the concentration of hydroxytyrosol did not differ statistically between the EVOOs (*p* = 0.380). As for tyrosol, during 2016–2017, the average calculated concentrations were 9.67 mg/kg in EVOOs from low altitude, 10.7 mg/kg in those originating from medium altitude, and 15.2 mg/kg in EVOOs from higher altitude, respectively. The statistical analysis showed that the concentration of tyrosol did not differ significantly in EVOOs originating from cultivars from different altitudes. During 2017–2018, the average concentration of tyrosol was approximately the same for the three zones, specifically, 2.55 mg/kg in EVOOs from low altitude, 3.08 mg/kg in EVOOs from medium altitude, and 2.67 mg/kg in EVOOs from high altitude, and there were no statistically significant differences among the results (*p* = 0.780).

#### 2.2.2. Farming Type

Different growing potentially affects the phenolic composition of olives, and consequently intervenes in the quality properties of the produced EVOOs [12]. According to the literature, the results are controversial [8]. Despite general perception of consumers that organic products are richer in nutrients [42,43], some works have reported differences in the concentration levels of individual phenols [17], and no significant differences in the average phenolic content [44], while others report higher phenolic concentration on olive oils from organic farming systems [43,45,46]. The effect of conventional and organic farming types on olive fruits and the produced EVOOs constitutes a major scientific issue and a critical topic of generalized discussion. For this reason, the phenolic content of the analyzed EVOOs was compared with respect to the type of farming.

**Figure 5:**
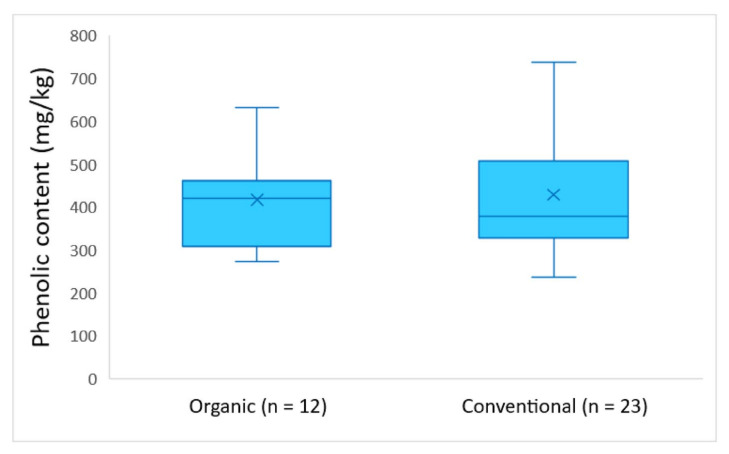
Phenolic content of the analyzed EVOOs produced in 2016–2017 in comparison with the farming type (organic or conventional).

According to Figure 5, the EVOOs produced during the harvesting period 2016–2017 had similar average phenolic content for both types of cultivar (417 mg/kg for organic and 429 mg/kg for conventional). In addition, the difference between organic and conventional cultivars was not statistically significant, as ANOVA showed a *p*-value > 0.05 (*p* = 0.78).

**Figure 6:**
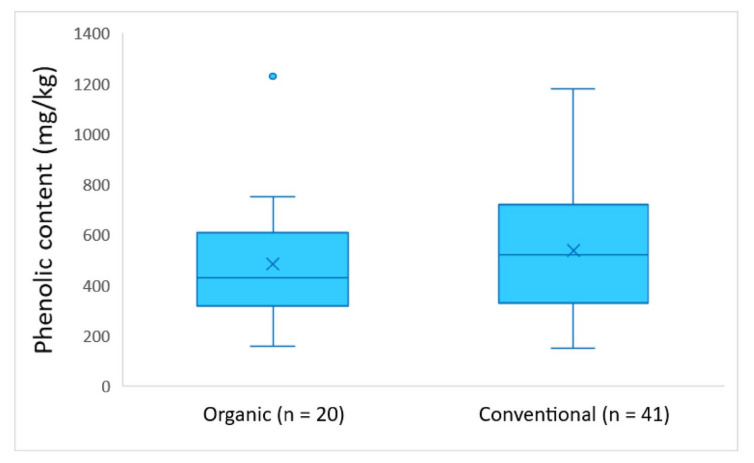
Phenolic content of the analyzed EVOOs produced in 2017–2018 in comparison with the farming type (organic or conventional).

On the basis of Figure 6, the olive oils from the conventional cultivar had a slightly higher average phenolic content (538 mg/kg) compared to the olive oils from the organic cultivar (483 mg/kg) during the harvesting period 2017–2018. However, the results of ANOVA showed that there was no statistically significant difference between the two types of cultivar (*p* = 0.43).

The results of this work indicate no significant differences in the average phenolic content between EVOOs grown with organic and conventional farming. Specifically, the average phenolic content was equal to 417 mg/kg for organic, and 429 mg/kg for conventional EVOOs, respectively, harvested in 2016–2017. The average phenolic content in EVOOs produced in the following harvesting year, 2017–2018, did not present any significant differences, either (organic EVOOs: 483 mg/kg; and conventional EVOOs: 538 mg/kg).

Τhe *p*-value for the interaction between altitude and farming type with two-way ANOVA proved that there was no statistically significant interaction between the two factors and the phenolic content (*p*-value > 0.05).

The average quantification results of hydroxytyrosol and tyrosol were equal to 12.0 mg/kg and 11.0 mg/kg in organic cultivars during the crop year 2016–2017, and 3.88 mg/kg and 2.85 mg/kg during the crop year 2017–2018, respectively. As for conventional cultivars, the average concentration of tyrosol was equal to 11.5 mg/kg in EVOOs produced in 2016–2017, and 2.84 mg/kg in EVOOs produced 2017–2018. The average concentration of hydroxytyrosol was equal to 18.5 mg/kg during 2016–2017, and 3.04 mg/kg during 2017–2018. The ANOVA analysis showed that there was no statistically significant difference between the concentration of hydroxytyrosol and tyrosol and the type of farming for both crop years (*p*-value > 0.05, in all cases).

Overall, the total sum of all the target and suspect phenolic compounds ranged between 237 and 737 mg/kg, with an average value of 425 mg/kg for the EVOOs produced during 2016–2017, and between 151 and 1230 mg/kg for the EVOOs produced during 2017–2018, with an average value of 522 mg/kg. The increase in the average concentration and sum between the two crop years could be associated with changes in the weather conditions, and especially temperature and rainfall (since the analyzed samples were produced in cultivars that were naturally watered by rain) within the two crop years. These findings suggest that further analysis should be carried out to evaluate the effects of weather conditions on the phenolic content between different crop years.

## 3. Materials and Methods

### 3.1. Chemicals and Standards

Methanol (LC-MS grade) and sodium hydroxide (>99%) were acquired from Merck (Darmstadt, Germany). Ammonium acetate (≥99%) and formic acid (LC-MS Ultra) were purchased from Fluka (Buchs, Switzerland). Isopropanol was obtained from Fisher Scientific (Geel, Belgium). Ultrapure water was obtained using a Milli-Q purification system (Millipore Direct-Q UV, Bedford, MA, USA).

Syringic acid 95% was purchased from Extrasynthèse (Genay, France). Gallic acid 98%, ferulic acid 98%, epicatechin 97%, p-coumaric 98%, oleuropein 98%, homovanillic acid 97%, syringaldehyde 98%, and pinoresinol 95% were purchased from Sigma-Aldrich (Steinheim, Germany). Hydroxytyrosol 98% and luteolin 98% were acquired from Santa Cruz Biotechnologies. Caffeic acid 99%, vanillin 99%, apigenin 97%, and tyrosol 98% were purchased from Alfa Aesar (Karlsruhe, Germany). Stock standard solutions of each analyte (1000 mg/L) were solubilized in methanol and stored at −20 °C in dark brown glass bottles. Mixed standard working solutions were prepared every laboratory day by appropriate dilution of the stock solutions with methanol:water (80:20, *v/v*) in the concentration range of 0.1–12 mg/L.

### 3.2. Instrumentation

A UHPLC system with an HPG-3400 pump (Dionex UltiMate 3000 RSLC, Thermo Fisher Scientific, Germany) coupled to a QTOF mass spectrometer (Maxis Impact, Bruker Daltonics, Bremen, Germany) was used for the analysis. Separation was carried out using an Acclaim RSLC C18 column (2.1 × 100 mm, 2.2 μm) purchased from Thermo Fisher Scientific (Driesch, Germany) with an ACQUITY UPLC BEH C18 pre-column (1.7 μm, VanGuard precolumn, Waters, Ireland). The column temperature was set at 30 °C. The mobile phase consisted of (A) 90% water, 10% methanol and 5 mM CH_3_COONH_4_, (B) 100% methanol and 5 mM CH_3_COONH_4_. The following gradient program was used: starting with 1% of B and a flow rate of 0.2 mL/min for 1 min, gradually increasing to 39% in the next 2 min, and then increasing to 99.9% and a flow rate of 0.4 mL/min for the following 11 min. These conditions remained constant for 2 min (flow rate 0.48 mL/min) and then the initial conditions (99% A, 1% B) were restored within 0.1 min (the flow rate decreased to 0.2 mL/min) for re-equilibration of the column.

The QTOF MS system was equipped with an electrospray ionization (ESI) interface, operating in a negative mode with the following settings: capillary voltage of 3500 V, end plate offset of 500 V, nebulizer pressure of 2 bar (N_2_), drying gas flow rate of 8 L/min (N_2_) and drying temperature of 200 °C. External calibration was performed daily with a sodium formate cluster solution consisting of 10 mM sodium formate in a mixture of isopropanol:water (1:1, *v*/*v*). Additionally, the calibration solution was injected at the beginning of each run, and a segment (0.1–0.25 min) in every chromatogram was used for internal calibration. Full scan mass spectra were recorded in the range from 50 to 1000 *m*/*z*, with a scan rate of 2 Hz. MS/MS experiments were conducted using data dependent acquisition (AutoMS, otofControl, Bruker Daltonics, Bremen, Germany) mode based on the fragmentation of the five most abundant precursor ions per scan. The instrument provided a typical resolving power (full width at half maximum) between 36,000 and 40,000 at *m*/*z* 226.1593, 430.9137, and 702.8636.

### 3.3. Sampling

**Figure 7:**
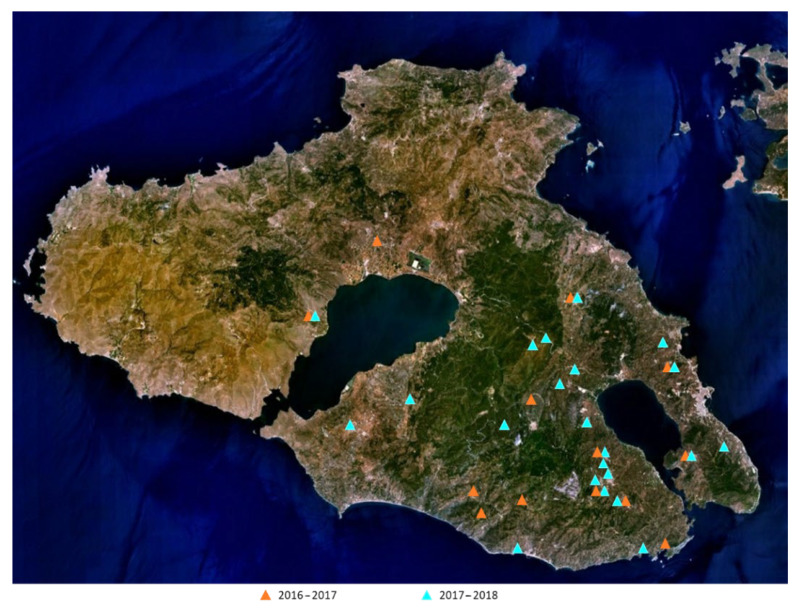
Geographical origin of EVOOs from the two harvesting seasons 2016–2017 and 2017–2018.

EVOOs of the *Kolovi* variety were collected from Lesvos island during the harvesting periods 2016–2017 (*n* = 35) and 2017–2018 (*n* = 62). The EVOOs were acquired from various locations of Lesvos, which are presented in Figure 7. The selected samples originated from cultivars that were not irrigated and no fertilizers were used, either. The altitudes at which the olive trees were cultivated and the types of farming (organic, conventional) were different among the samples, while all EVOOs were produced with a two-phase decanter, and stored in amber glass bottles at 4 °C until analysis. Table S5 presents more information about the EVOOs for the two harvest years (territory, altitude, type of farming).

### 3.4. Sample Preparation

**Figure 8:**
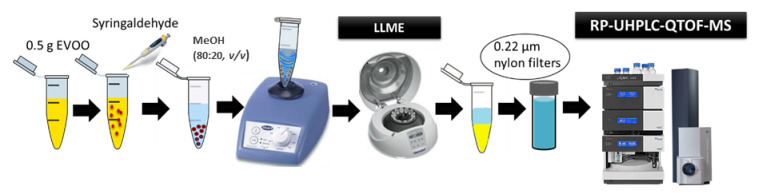
Schematic representation of the LLME-RP-UHPLC-QTOF-MS methodology applied in the analysis of EVOOs.

A liquid–liquid micro-extraction (LLME) method was used in order to isolate the phenolic compounds from the olive oil samples [26]. The LLME protocol applied in the analysis of EVOOs is schematically illustrated in Figure 8. In brief, 0.5 g (± 0.005) of sample was weighed in a 2 mL Eppendorf tube and spiked with 1.3 mg/L internal standard. For the extraction, 0.5 mL of methanol:water (80:20, *v*/*v*) was added. Then, the mixture was vortexed for 2 min and centrifuged for 5 min at 13,400 rpm. In the next step, the upper phase was collected and filtered through membrane syringe filter of regenerated cellulose (CHROMAFIL^®^ RC) (15 mm diameter, 0.22 µm pore size, purchased from Macherey-Nagel, Düren, Germany). The extracts were stored at −80 °C prior to analysis. Finally, 5 µL of this solution was injected into the chromatographic system.

### 3.5. Quality Control

Quality control (QC) samples were used to ensure that the analytical system was stable during analysis. A QC sample was prepared by mixing aliquots of all samples according to Want et al. [63], and was spiked with a standard solution mixture (1 mg/L) that comprised apigenin, gallic acid, hydroxytyrosol, oleuropein, and tyrosol. The QC sample was used to stabilize the analytical system prior to the analysis of the main batch of samples and assess its performance. In the beginning of the analysis, the QC sample was injected 5 times for conditioning and then, it was injected every 10 sample injections throughout the analytical run to provide a set of data and inspect the performance of the analytical system. Procedural blanks were also prepared and processed in the chromatographic system to detect any potential contamination. The quality control results are presented in Table S6. The %RSD areas of the standard compounds ranged between 2.57–4.46% (*n* = 11). The t_R_ shift was in the range 0.04–0.08% (*n* = 11), and the mass error was less than 0.16 mDa, confirming the good performance of the analytical system.

### 3.6. Fingerprinting Strategies

#### 3.6.1. Target Screening

The applied RP-UHPLC-ESI-QTOF-MS/MS methodology has been previously validated and proved suitable for identification and quantification purposes [26]. For the identification of target analytes, a target screening workflow was applied. Target screening is based on the determination of analytes using standard solutions for confirmation [64]. A reference standard is necessary to compare and match the experimental t_R_ and the MS/MS fragments. A target list was created from the literature including 14 significant phenolic compounds that have already been identified in VOOs/EVOOs. The list consisted of different classes of compounds, such as caffeic acid, ferulic acid, gallic acid, homovanillic acid, p-coumaric acid and syringic acid from the class of phenolic acids; hydroxytyrosol and tyrosol from phenolic alcohols; vanillin from the class of phenolic aldehydes; apigenin, epicatechin and luteolin from the class of flavonoids; oleuropein from the class of secoiridoids; and the lignan pinoresinol. The initial target list with the molecular formulas of the target compounds, their calculated molecular ions in negative ESI, and t_R_s can be found in Table S7.

Target screening was followed using Bruker software packages (Bruker Daltonics, Bremen, Germany) TASQ 1.4 and DataAnalysis 4.3 in combination with other tools available in these packages, such as SmartFormula Manually and Bruker Compass Isotope Pattern. EICs were obtained according to the following parameters: mass accuracy window up to 2.5 mDa, isotopic fit below or equal to 100 mSigma (mSigma value is a measure for the goodness of fit between measured and theoretical isotopic pattern), signal-to-noise (S/N) threshold was set at 3, minimum peak area threshold was set at 2000 and minimum ion intensity threshold was set at 500. Relative tolerance of the t_R_ window was set lower than ±0.2 min. The target analytes were identified on the basis of mass accuracy, retention time, isotopic pattern and MS/MS fragments.

#### 3.6.2. Suspect Screening

A suspect list generated from the literature including all the phenolic compounds that have already been identified in VOOs/EVOOs and different organs of *Olea europaea* L. in a previous study of our group [26], was used to scan the potential presence of 95 phenolic compounds in the analyzed samples. The suspect list is presented in Table S3. Similar to target screening, Bruker software packages (Bruker Daltonics, Bremen, Germany) TASQ 1.4 and DataAnalysis 4.3 were used for suspect analysis. The masses of the deprotonated ions were calculated based on their molecular formulas and EICs were created using the following parameters: mass accuracy threshold up to 2.5 mDa, isotopic fit below or equal to 100 mSigma, minimum peak area threshold was set to 3000 and minimum ion intensity threshold was set to 800. The suspect identification workflow incorporated strict filtering steps, interpretation of the MS/MS spectra and t_R_ prediction, as rpeviosly described by Kalogiouri et al. [26,50]. The MS/MS fragments were interpreted using Metfrag and compared with the fragments and the experimental t_R_s reported in previous works of our group [24,26,50].

### 3.7. Statistical Analysis

Statistical analysis was performed using one-way ANOVA from Data Analysis tool of Microsoft Excel (Microsoft, WA, USA) at a 95% confidence level. In addition, two-way ANOVA was applied to evaluate if there were significant statistical differences between all factors (altitude and farming type) and the phenolic content of the analyzed samples, comparing the quantification and semi-quantification results at a 95% confidence level.

## 4. Conclusions

This study contributes to the field of olive oil authenticity and traceability with the introduction of a LLME-RP-UHPLC-QTOF-MS analytical methodology employing target and suspect screening. Ninety-seven monovarietal *Kolovi* EVOOs produced during the harvesting years 2016–2017 and 2017–2018 were analyzed and five phenolic compounds were identified with target screening, and 20 with suspect screening. The target analytes were quantified based on their commercially available reference standards, and the suspect compounds were semi-quantified on the basis of target compounds having similar structures.

The calculation of the olive oil polyphenols supporting the EC Reg. 432/2012 ranged between 130 and 1218 mg/kg, indicating that 78% of the analyzed monovarietal *Kolovi* EVOOs could be labeled with the Health Claim “contributing to the protection of blood lipids from oxidative stress”. The total sum of all the target and suspect phenolic compounds ranged between 237 and 737 mg/kg, with an average value of 425 mg/kg for the EVOOs produced during 2016–2017, and between 151 and 1230 mg/kg for the EVOOs produced during 2017–2018, with an average value of 522 mg/kg. The high phenolic content highlights the high nutritional value and several health-related properties related to the consumption of the *Kolovi* EVOOs. The identified phenolic compounds demonstrate favorable health effects in relation to oxidative stress and antithrombotic profiles, cardiovascular risk factors, acting against chronic diseases such as cancer, obesity, diabetes, acting against Alzheimer’s disease, exhibiting hypotensive, antimicrobial and anti-inflammatory properties. These findings strongly suggest that *Kolovi* EVOOs, beyond adequate nutrition, improve health and promots well-being.

Furthermore, the evaluation of the agronomical factors indicated that the phenolic content of the EVOOs is strongly dependent on the altitude. The *Kolovi* EVOOs originating from medium (100–300 m) and high altitude (300–600 m), exhibited a significantly higher phenolic content compared to those grown in territories below 100 m. Finally, the effect of the type of farming on the phenolic content was also evaluated, and the results indicated that there is not significant statistical difference in the phenolic concentration levels between organic and conventional EVOOs.

## Figures and Tables

**Table 1:** Target screening results.

Compound	Molecular Formula	[M–H]^−^ *m*/*z* Theoretical	[M–H]^−^ *m*/*z* Experimental	t_R_ Standard (min)	Δt_R_ (min)
Phenolic alcohols					
Hydroxytyrosol	C_8_H_10_O_3_	153.0557	153.0557	3.53	−0.01
Tyrosol	C_8_H_10_O_2_	137.0608	137.0608	4.07	+0.02
Flavonoids					
Apigenin	C_15_H_10_O_5_	269.0455	269.0455	8.24	−0.03
Luteolin	C_15_H_10_O_6_	285.0404	285.0405	7.55	−0.04
Lignans					
Pinoresinol	C_20_H_22_O_6_	357.1343	357.1341	6.49	+0.01

**Table 2:** Suspect screening results.

Compound	Molecular Formula	[M–H]^−^ *m*/*z* Calculated	[M–H]^−^ *m*/*z* Experimental	Fragments *m*/*z*	Elemental Formula	t_R_ (min)
Hydroxytyrosol acetate	C_10_H_12_O_4_	195.0663	195.0663	134.0373 149.0608 161.0246	C_8_H_6_O_2_ C_9_H_9_O_2_ C_9_H_5_O_3_	6.70
Decarboxymethyl lingstroside aglycone (Oleocanthal)	C_17_H_20_O_5_	303.1237	303.1236	124.0532 137.0605 147.0450 165.0551 183.0662	C_7_H_8_O_2_ C_8_H_10_O_2_ C_9_H_7_O_2_ C_9_H_9_O_3_ C_9_H_11_O_4_	6.43
Decarboxymethyl oleuropein aglycone (Oleacein)	C_17_H_20_O_6_	319.1187	319.1185	69.0342 95.0501 123.0448 139.0602 165.0556 183.0660 195.0656	C_4_H_5_O C_6_H_7_O C_7_H_7_O_2_ C_8_H_11_O_2_ C_9_H_9_O_3_ C_9_H_11_O_4_ C_10_H_11_O_4_	5.60
10-Hydroxy-10-methyl oleuropein aglycone	C_20_H_24_O_9_	407.1347	407.1347	99.0453 111.0087 121.0295 135.0453 137.0243 149.0245 163.0402 179.0351 195.0665 241.0871	C_5_H_7_O_2_ C_5_H_3_O_3_ C_7_H_5_O_2_ C_8_H_7_O_2_ C_7_H_5_O_3_ C_8_H_5_O_3_ C_9_H_7_O_3_ C_9_H_7_O_4_ C_10_H_11_O_4_ C_15_H_13_O_3_	6.71
10-Hydroxy-decarboxymethyl oleuropein aglycone	C_17_H_20_O_7_	335.1136	335.1135	59.0139 85.0296 121.0292 151.0401 153.0557 155.0716 199.0613	C_2_H_3_O_2_ C_4_H_5_O_2_ C_7_H_5_O_2_ C_8_H_7_O_3_ C_8_H_9_O_3_ C_8_H_11_O_3_ C_9_H_11_O_5_	4.30
10-Hydroxy oleuropein aglycone	C_19_H_22_O_9_	393.1191	393.1190	137.0244 181.0502	C_7_H_5_O_3_ C_9_H_9_O_4_	4.82
Lingstroside aglycone	C_19_H_22_O_7_	361.1291	361.1291	259.0975 291.0875	C_15_H_15_O_4_ C_15_H_15_O_6_	6.63
Lingstroside aglycone monoaldehydic form	C_19_H_22_O_7_	361.1291	361.1291	137.0608 241.0718	C_8_H_9_O_2_ C_11_H_13_O_6_	7.84
Lingstroside aglycone dialdehydic form	C_19_H_22_O_7_	361.1291	361.1291	69.0346 101.0244 259.0976	C_4_H_5_O C_4_H_5_O_3_ C_15_H_15_O_4_	8.15
Oleokoronal	C_19_H_22_O_7_	361.1291	361.1291	195.0663 291.0874	C_10_H_11_O_4_ C_15_H_15_O_6_	8.34
Methyl oleuropein aglycone	C_20_H_24_O_8_	391.1398	391.1396	59.0140 67.0192 99.0456 111.0086 137.0608 291.0875	C_2_H_3_O_2_ C_4_H_3_O C_5_H_7_O_2_ C_5_H_3_O_3_ C_8_H_9_O_2_ C_16_H_15_O_6_	7.49
Oleuropein aglycone	C_19_H_22_O_8_	377.1241	377.1242	111.0088 149.0244 195.0645 275.0918 307.0823	C_5_H_3_O_3_ C_8_H_5_O_3_ C_10_H_11_O_4_ C_15_H_15_O_5_ C_15_H_15_O_7_	7.30
Oleuropein aglycone monoaldehydic form	C_19_H_22_O_8_	377.1241	377.1242	69.0345 99.0088 121.0294 127.0400	C_4_H_5_O C_4_H_3_O_3_ C_7_H_5_O_2_ C_6_H_7_O_3_	7.43
Oleuropein aglycone dialdehydic form	C_19_H_22_O_8_	377.1241	377.1242	59.0139 67.0187 95.0138 123.0453 128.0478 153.0558 195.0662	C_2_H_3_O_2_ C_4_H_3_O C_5_H_3_O_2_ C_7_H_7_O_2_ C_6_H_8_O_3_ C_8_H_9_O_3_ C_10_H_11_O_4_	7.62
Oleomissional	C_19_H_22_O_8_	377.1241	377.1242	101.0245 163.0400	C_4_H_5_O_3_ C_9_H_7_O_3_	7.76
1-Acetoxypinoresinol	C_22_H_24_O_8_	415.1398	415.1397	151.0402 280.0951 343.1188	C_8_H_7_O_3_ C_14_H_16_O_6_ C_19_H_19_O_6_	6.40
1-Hydroxypinoresinol	C_20_H_22_O_7_	373.1292	373.1290	121.0294 151.0401 163.0402	C_7_H_5_O_2_ C_8_H_7_O_3_ C_9_H_7_O_3_	6.38
Syringaresinol	C_22_H_26_O_8_	417.1554	417.1557	127.0406 181.0505	C_6_H_7_O_3_ C_9_H_9_O_4_	6.19
Elenolic acid	C_11_H_14_O_6_	241.0717	241.0716	59.0137 95.0496 127.0400 151.0402 171.0300	C_2_H_3_O_2_ C_6_H_7_O C_6_H_7_O_3_ C_8_H_7_O_3_ C_7_H_7_O_5_	4.51
Hydroxylated form of elenolic acid	C_11_H_14_O_7_	257.0667	257.0663	59.0104 137.0603 181.0535	C_2_H_3_O_2_ C_8_H_9_O_2_ C_9_H_9_O_4_	1.37

## Data Availability

Not available.

## Supplementary Materials

The following are available online, Figure S1: EIC of lingstroside transformation to: lingstroside aglycone (1); lingstroside aglycone monoaldehydic form (2); lingstroside aglycone dialdehydic form (3); oleokoronal (4), Figure S2: EIC of οleuropein transformation to: oleuropein aglycone (1); oleuropein aglycone monoaldehydic form (2); oleuropein aglycone dialdehydic form (3); oleomissional (4), Figure S3: Characteristic spectra of: (a) oleuropein aglycone; (b) oleuropein aglycone monoaldehydic form; (c) oleuropein aglycone dialdehydic; (d) oleomissional, Table S1: Standard calibration curves, Table S2: Target screening quantification results (mg/kg), Table S3: Suspect list, Tables S4: Suspect screening quantification results (mg/kg), Table S5: Geographical region, altitude, type of farming and harvesting period of the Kolovi EVOOs, Table S6: Quality Control Results, Table S7: Target list.

## Samples Availability

Samples of the compounds are not available from the authors.
