# Supplementary material for: Characterization of the Phenolic Fingerprint of Kolovi Extra Virgin Olive Oils from Lesvos with Regard to Altitude and Farming System Analyzed by UHPLC-QTOF-MS
Source: Molecules. 2021 Sep 17;26(18):5634. doi: 10.3390/molecules26185634 (PMC8471387; doi:10.3390/molecules26185634)
Supplement: Supplementary file 1 [file molecules-26-05634-s001.zip › molecules-1332879-supplementary.pdf]

Supplementary Material

Characterization of the phenolic fingerprint of *Kolovi* extra virgin olive oils from Lesvos with regard to altitude and farming system analyzed by UHPLC-QTOF-MS

Natasa P. Kalogiouri, Evangelia Kritikou, Ioannis Martakos, Constantina Lazarou, Michalis Pentogennis and Nikolaos S. Thomaidis

Table S1. Standard calibration curves.

Compound	Equation $y = b (\pm S_b) x + a (\pm S_a)$ (linear range: 0.1 – 12 mg/kg)	r^2
**Hydroxytyrosol**	y = 3.40 (±0.10) x - 0.84 (±0.63)	0.997
**Oleuropein**	y = 0.903 (±0.013) x - 0.102 (±0.082)	0.999
**Tyrosol**	y = 0.719 (±0.017) x + 0.03 (±0.10)	0.998
**Apigenin**	y = 9.73 (±0.19) x +15.0 (±1.2)	0.998
**Luteolin**	y = 6.57 (±0.12) x - 1.44 (±0.76)	0.999
**Pinoresinol**	y = 0.2059 (±0.0033) x - 0.020 (±0.020)	0.999

Table S2. Target screening quantification results (mg/kg).

Sample	Hydroxytyrosol (mg/kg)	Tyrosol (mg/kg)	Apigenin (mg/kg)	Luteolin (mg/kg)	Pinoresinol (mg/kg)
ME1	11.7	12.1	0.15	1.90	0.78
MO1	nd	11.8	0.26	0.22	0.52
MO3	11.6	11.4	0.85	1.55	0.6
PA1	11.7	12.0	0.15	1.63	0.57
PL1	11.6	12.0	0.32	5.43	0.62
PA2	11.6	11.7	0.14	1.88	0.5
AG1	11.5	11.7	0.16	2.05	0.65
GE1	nd	nd	0.18	5.21	nd
ME2	35.2	35.1	0.29	2.18	1.19
PR1	58.6	46.5	0.30	0.72	1.11
PA3	35.3	34.8	0.85	0.42	1.61
PL3	59.0	34.7	nd	1.07	1.37
PH1	nd	nd	0.21	0.24	0.75
AK1	11.9	11.9	0.76	0.63	0.76
PH2	12.6	12.3	0.87	2.53	0.99
PH3	12.3	12.1	0.91	1.56	0.86
GE2	11.7	12.4	0.89	2.14	0.82
GE3	12.2	12.1	0.82	1.9	0.82
SK1	11.9	12.4	0.82	1.24	1.21
DF1	12.5	0.47	nd	1.14	0.82
GE4TH	12.5	0.52	11.9	0.91	0.77
KO1	12.8	0.5	12.4	0.84	0.66
KO3	11.5	0.48	13.9	1.11	0.55
KO4	11.7	0.45	11.8	0.77	0.62
KP1	12.4	0.44	11.9	0.85	0.78
GE4	12.8	0.57	11.9	0.92	0.91
GE5	12.5	0.7	11.8	0.85	0.64
GE6	12.2	0.47	11.7	1.21	0.45

Sample	Hydroxytyrosol (mg/kg)	Tyrosol (mg/kg)	Apigenin (mg/kg)	Luteolin (mg/kg)	Pinoresinol (mg/kg)
KA1	12.2	0.5	12.2	0.75	0.74
PL4	12.3	0.41	12.2	0.82	0.63
M1	12.6	12.4	0.85	4.58	0.91
M2	12.9	12.4	0.92	4.75	1.07
M3	11.7	12.4	0.71	4.53	0.82
M4	11.8	12.2	0.74	4.61	0.87
EE1	11.7	12.7	0.94	4.63	0.95
1087ELSG	0.44	2.76	2.58	2.55	2.21
1248ELOA	7.65	3.92	2.93	2.12	4.83
1298ELGP	4.13	1.61	2.31	0.43	4.36
2039ELET	1.03	3.08	2.85	2.75	8.70
2040LAZF	0.44	1.67	2.59	1.72	6.29
2042LAIX	0.40	4.30	3.04	3.92	6.81
2051LAPK	0.96	4.15	2.47	1.71	5.10
2052LATHG	2.84	5.84	2.09	1.01	3.34
2057LASI	0.53	1.14	2.40	2.11	3.95
2085ELGG	0.38	0.83	2.56	2.52	1.92
2093ELBB	0.97	1.29	2.46	1.98	4.99
2108ELEK	0.71	1.36	2.79	2.34	6.94
2112ELNK	0.62	0.70	3.13	4.61	7.23
2135LAEA	2.89	1.18	2.60	2.34	2.68
2136LAPK	5.03	3.19	2.70	1.49	6.58
2137LAMS	12.9	16.0	2.23	0.51	4.98
2138LATHM	3.81	1.78	3.21	4.80	4.23
2139LAOT	10.1	4.28	2.78	3.08	3.47
2140LANX	1.20	0.83	2.63	2.29	2.34
2154ELME	1.26	1.10	3.03	1.32	2.99
2155ELET	2.13	1.34	2.25	0.97	2.43
2169ELEK	1.52	3.03	2.26	1.28	2.29
2170ELGK	0.28	1.46	2.40	2.25	3.46
2178LAOX	6.18	3.45	2.57	1.38	1.97
2179LAKZ	6.02	3.93	2.45	0.95	1.90
2180LAGA	1.48	3.42	3.14	2.68	3.10
2181LAKA	1.63	2.44	2.81	1.40	1.99
2182LAID	2.09	3.15	2.09	0.55	0.80
2183LADK	2.46	2.51	2.98	2.60	1.96
2225LAPT	12.3	3.97	2.62	2.98	4.28
2227ELNK	1.62	0.36	2.53	2.92	2.92
2228ELEK	3.93	0.67	2.40	2.19	3.43
2238ELMK	0.99	0.41	2.29	0.85	1.68
2239ELND	2.75	1.11	2.36	0.66	1.38
2242LAPK	1.26	0.65	2.64	2.16	4.42
2243LAPK	2.64	3.68	3.50	3.39	7.53
2251ELTHS	2.95	0.98	2.67	1.82	3.96

Sample	Hydroxytyrosol (mg/kg)	Tyrosol (mg/kg)	Apigenin (mg/kg)	Luteolin (mg/kg)	Pinoresinol (mg/kg)
2252ELXX	1.74	1.14	3.54	1.62	6.76
2278ELPA	0.99	1.03	2.92	2.32	4.25
2290ELMK	2.89	1.52	3.16	3.21	6.43
2291ELLX	0.62	0.84	3.20	3.83	7.18
2293LAXG	4.22	3.76	4.42	7.71	9.63
2309ELXX	0.84	2.24	3.60	1.93	11.5
2345LAMM	2.31	3.82	3.50	3.29	5.33
2346LANE	13.3	7.08	3.49	3.83	7.74
2349ELGK	3.45	2.80	2.31	0.69	3.02
3014KAAK	0.19	0.99	2.35	1.14	2.53
3095KANK	0.82	0.57	2.34	1.77	2.13
3355KADL	5.89	3.53	2.56	1.22	5.84
4292PAGPS	27.4	9.11	2.31	0.56	3.98
4302PAKA	3.25	1.38	2.69	1.84	3.04
6356PAGG	0.15	1.88	2.81	2.42	5.97
7034PLXP	0.36	2.37	2.67	1.59	8.07
7046MAED	2.30	9.41	2.51	1.57	6.11
7070MASK	1.03	3.68	2.44	1.77	1.95
7097KADB	0.99	3.34	2.48	1.54	4.64
7099PLDF	2.37	4.18	2.74	4.62	2.06
7109PLDP	0.86	2.82	3.19	3.69	6.41
7144PLAB	3.71	3.64	2.72	3.82	2.86
7153MASK	1.72	1.48	2.64	3.19	1.84
7198MANM	10.7	4.17	2.91	2.18	1.86
7280KALN	1.03	1.46	2.92	2.36	4.73

nd: not detected

Table S3. Suspect list.

**Compound**	**Molecular Formula**	**[M-H]$^-$ *m/z* calculated**	**SMILES**
1-(3′-methoxy-4′hydroxy)Phenyl-6.7-dihydroxy-isochroman	$C_{16}H_{16}O_5$	287.0924	COC1=C(O)C=CC(=C1)C1=C(O)C(O)=CC2=C1COCC2
10-Hydroxy decarboxymethyl oleuropein aglycone	$C_{17}H_{20}O_7$	335.1150	[H]C(O)\C=C1/[C@H](O)OC=C([H])[C@H]1CC(=O)OCCC1=CC(O)=C(O)C=C1
10-Hydroxy oleuropein aglycone	$C_{19}H_{22}O_9$	393.1193	[H]C(O)\C=C1/[C@H](O)OC=C([C@H]1CC(=O)OCCC1=CC(O)=C(O)C=C1)C(=O)OC
10-Hydroxy-10Methyl oleuropein aglycone	$C_{20}H_{24}O_9$	407.1347	COC(=O)C1=CO[C@@H](O)\C(=C/C(C)O)[C@@H]1CC(=O)OCCC1=CC(O)=C(O)C=C1
1-Acetoxypinoresinol	$C_{22}H_{24}O_8$	415.1398	COC1=C(O)C=CC(=C1)C1OCC2(OC(C)=O)C1COC2C1=CC(OC)=C(O)C=C1
1-Hydroxypinoresinol	$C_{20}H_{22}O_7$	373.1292	COC1=C(O)C=CC(=C1)C1OCC2(O)C1COC2C1=CC(OC)=C(O)C=C1
1-Phenyl-6.7-dihydroxy-isochroman	$C_{15}H_{14}O_3$	241.0870	OC1=C(O)C=C2C(OCCC2=C1)C1=CC=CC=C1
2''-Methoxy-(R)-oleuropein	$C_{26}H_{34}O_{14}$	569.1875	COC(COC(=O)CC1\C(=C/C)C(OC2OC(CO)C(O)C(O)C2O)OC=C1C(=O)OC)C1=CC(O)=C(O)C=C1
2-Methoxyoleuropein	$C_{26}H_{34}O_{14}$	569.1875	[H][C@]1(CC(=O)OC[C@@H](OC)C2=CC(O)=C(O)C=C2)\C(=C/C)[C@H](O[C@@H]2O[C@H](CO)[C@@H](O)[C@H](O)[C@H]2O)OC=C1C(=O)OC
2-Vicenin	$C_{27}H_{30}O_{15}$	593.1511	c1cc(ccc1c2cc(=O)c3c(c(c(c(c3o2)[C@H]4[C@@H]([C@H]([C@@H]([C@H](O4)CO)O)O)O)O)[C@H]5[C@@H]([C@H]([C@@H]([C@H](O5)CO)O)O)O)O)O
5-[2-(3.4-Dihydroxyphenyl)ethyl] 1-methyl (2S.3R)-2-(dihydroxymethyl)-3-[(2E)-1-oxo-2-buten-2-yl]pentanedioate	$C_{19}H_{24}O_9$	395.1347	[H]\C(C)=C(/C([H])=O)C([H])(CC(=O)OCCC1=CC=C(O)C(O)=C1)[C@]([H])(C(=O)OC)C([H])(O)O
8-Acetoxy-4'-methoxypinoresinol	$C_{23}H_{26}O_8$	429.1554	COC1=C(O)C=CC(=C1)C1OCC2C(OCC12OC(C)=O)C1=CC(OC)=C(OC)C=C1

Compound	Molecular Formula	[M-H]- *m/z* calculated	SMILES
8-Acetoxypinoresinol	$C_{22}H_{24}O_8$	415.1398	COC1=C(O)C=CC(=C1)C1OCC2(OC(C)=O)C1COC2C1=CC(OC)=C(O)C=C1
8-Hydroxy-4'-methoxypinoresinol	$C_{21}H_{24}O_7$	387.1449	COC1=C(OC)C=C(C=C1)C1OCC2(O)C1COC2C1=CC(OC)=C(O)C=C1
8-Hydroxypinoresinol	$C_{20}H_{22}O_7$	373.1292	[H][C@]12CO[C@H](C3=CC(OC)=C(O)C=C3)[C@@]1(O)CO[C@@H]2C1=CC(OC)=C(O)C=C1
8-Hydroxypinoresinol 8-glucoside	$C_{26}H_{32}O_{12}$	535.1821	COC1=C(O)C=CC(=C1)C1OCC2(OC3OC(CO)C(O)C(O)C3O)C1COC2C1=CC(OC)=C(O)C=C1
Absisic acid	$C_{15}H_{20}O_4$	263.1288	C\C(\C=C\[C@@]1(O)C(C)=CC(=O)CC1(C)C)=C\C(O)=O
Aesculetin	$C_9H_6O_4$	177.0193	c1cc(=O)oc2c1cc(c(c2)O)O
Aesculin	$C_{15}H_{16}O_9$	339.0721	OC[C@H]1O[C@@H](OC2=C(O)C=C3OC(=O)C=CC3=C2)[C@H](O)[C@@H](O)[C@@H]1O
Apigenin-7-glucoside	$C_{21}H_{20}O_{10}$	431.0983	OC[C@H]1O[C@@H](OC2=CC(O)=C3C(=O)C=C(OC3=C2)C2=CC=C(O)C=C2)[C@H](O)[C@@H](O)[C@@H]1O
Benzoic acid	$C_7H_6O_2$	121.0295	OC(=O)C1=CC=CC=C1
Berchemol	$C_{20}H_{42}O_7$	393.2857	COC1=CC(C[C@@H]2CO[C@H](C3=CC(OC)=C(O)C=C3)[C@]2(O)CO)=CC=C1O
b-hydroxytyrosol ester of methyl malate	$C_{13}H_{16}O_7$	283.0823	COC(=O)C(O)CC(=O)OCCC1=CC=C(O)C(O)=C1
Calceolarioside	$C_{23}H_{26}O_{11}$	477.1402	OC[C@H]1O[C@@H](OCCC2=CC=C(O)C(O)=C2)[C@H](O)[C@@H](O)[C@@H]1OC(=O)\C=C\C1=CC(O)=C(O)C=C1
Campneoside	$C_{30}H_{38}O_{16}$	653.2087	COC(CO[C@H]1O[C@@H](CO)[C@H](OC(=O)\C=C\C2=CC=C(O)C(O)=C2)[C@@H](O[C@H]2O[C@H](C)[C@@H](O)[C@H](O)[C@@H]2O)[C@@H]1O)C1=CC(O)=C(O)C=C1
Chrysoeriol/ 3methyl-o-luteolin	$C_{16}H_{12}O_6$	299.0561	COC1=CC(=CC=C1O)C1=CC(=O)C2=C(O)C=C(O)C=C2O1
Cichoriin	$C_{15}H_{16}O_9$	339.0721	OCC1OC(OC2=C(O)C=C3C=CC(=O)OC3=C2)C(O)C(O)C1O

Compound	Molecular Formula	[M-H]⁻ *m/z* calculated	SMILES
Cinnamic acid	$C_9H_8O_2$	147.0451	OC(=O)\C=C\C1=CC=CC=C1
Cyanidin-3-glucoside	$C_{21}H_{21}O_{11}$	448.1011	OC[C@@H]1OC(OC2=CC3=C(O)C=C(O)C=C3[O+]=C2C2=CC=C(O)C(O)=C2)[C@@H](O)[C@H](O)[C@H]1O
Cyanidin-3-rutinoside	$C_{27}H_{31}O_{15}$	594.1590	C[C@@H]1O[C@@H](OC[C@H]2O[C@@H](OC3=CC4=C(C=C(O)C=C4O)[O]=C3C3=CC=C(O)C(O)=C3)[C@H](O)[C@@H](O)[C@@H]2O)[C@H](O)[C@H](O)[C@H]1O
Decarboxymethyl lingstroside aglycone	$C_{17}H_{20}O_5$	303.1237	C\C=C(\C=O)[C@@H](CC=O)CC(=O)OCCC1=CC=C(O)C=C1
Decarboxymethyl oleuropein aglycone	$C_{17}H_{20}O_6$	319.1185	C\C=C(\C=O)[C@@H](CC=O)CC(=O)OCCC1=CC(O)=C(O)C=C1
Demethyl oleuropein	$C_{24}H_{30}O_{13}$	525.1613	C\C=C1\[C@H](O[C@@H]2O[C@H](CO)[C@@H](O)[C@H](O)[C@H]2O)OC=C([C@H]1CC(=O)OCCC1=CC=C(O)C(O)=C1)C(O)=O
Diosmetin	$C_{16}H_{12}O_6$	299.0561	COC1=CC=C(C=C1O)C1=CC(=O)C2=C(O)C=C(O)C=C2O1
Diosmin	$C_{28}H_{32}O_{15}$	607.1668	COC1=CC=C(C=C1O)C1=CC(=O)C2=C(O)C=C(O[C@@H]3O[C@H](CO[C@@H]4O[C@@H](C)[C@H](O)[C@@H](O)[C@H]4O)[C@@H](O)[C@H](O)[C@H]3O)C=C2O1
Elenolic acid	$C_{11}H_{14}O_6$	241.0714	COC(=O)C1=COC(C)C(C=O)C1CC(O)=O
Elenolic acid glucoside	$C_{17}H_{24}O_{11}$	403.1245	COC(=O)C1=CO[C@@H](O[C@@H]2O[C@H](CO)[C@@H](O)[C@H](O)[C@H]2O)\C(=C\C)[C@@H]1CC(O)=O
Elenolic acid methyl ester	$C_{12}H_{16}O_6$	255.0874	COC(=O)C[C@H]1[C@H](C=O)[C@H](C)OC=C1C(=O)OC
Elenolide	$C_{11}H_{12}O_5$	223.0611	COC(=O)C1=COC(=O)CC1\C(=C\C)C=O
Eugenol	$C_{10}H_{12}O_2$	163.0764	COC1=C(O)C=CC(CC=C)=C1
Fraxamoside	$C_{25}H_{30}O_{13}$	537.1613	[H][C@@]12CC(=O)OC[C@H](OC[C@H]3O[C@@H](O[C@H](OC=C1C(=O)OC)\C2=C\C)[C@H](O)[C@@H](O)[C@@H]3O)C1=CC(O)=C(O)C=C1
Fustin	$C_{15}H_{12}O_6$	287.0561	c1cc(c(cc1[C@@H]2[C@H](C(=O)c3ccc(cc3O2)O)O)O)O

Compound	Molecular Formula	[M-H]- *m/z* calculated	SMILES
Gallocatechin	$C_{15}H_{14}O_7$	305.0666	O[C@@H]1CC2=C(O)C=C(O)C=C2O[C@H]1C1=CC(O)=C(O)C(O)=C1
Gentisic acid	$C_7H_6O_4$	153.0193	OC(=O)C1=CC(O)=CC=C1O
Guaiacol	$C_7H_8O_2$	123.0451	COc1ccccc1O
Hellicoside	$C_{29}H_{36}O_{17}$	655.1879	c1cc(c(cc1/C=C/C(=O)O[C@@H]2[C@H](O[C@H]([C@@H]([C@H]2O[C@H]3[C@@H]([C@@H]([C@@H]([C@H](O3)CO)O)O)O)O)OCC(c4ccc(c(c4)O)O)O)CO)O)O
Hesperidin	$C_{28}H_{34}O_{15}$	609.1824	COC1=CC=C(C=C1O)[C@@H]1CC(=O)C2=C(O)C=C(O[C@@H]3O[C@H](CO[C@@H]4O[C@@H](C)[C@H](O)[C@@H](O)[C@H]4O)[C@@H](O)[C@H](O)[C@H]3O)C=C2O1
Homovanillyl alcohol	$C_9H_{12}O_3$	167.0713	COC1=CC(CCO)=CC=C1O
Hydroxylated form of elenolic acid	$C_{11}H_{14}O_7$	257.0667	COC(=O)C1=CO[C@@H](CO)[C@@H](C=O)[C@@H]1CC(O)=O
Hydroxyphenylacetic acid	$C_8H_8O_3$	151.0400	OC(C(O)=O)C1=CC=CC=C1
Hydroxytyrosol 1-O-glucoside	$C_{14}H_{20}O_8$	315.1085	OCC1OC(OCCC2=CC(O)=C(O)C=C2)C(O)C(O)C1O
Hydroxytyrosol 3-O-beta-D-glucuronide	$C_{14}H_{18}O_9$	329.0878	OCCC1=CC(O[C@@H]2OC([C@@H](O)[C@H](O)C2O)C(O)=O)=C(O)C=C1
Hydroxytyrosol 4-O-glucoside	$C_{14}H_{20}O_8$	315.1085	OCCC1=CC(O)=C(OC2OC(CO)C(O)C(O)C2O)C=C1
Hydroxytyrosol acetate	$C_{10}H_{12}O_4$	195.0660	CC(=O)OCCC1=CC(O)=C(O)C=C1
Isoacteoside	$C_{29}H_{36}O_{15}$	623.1981	C[C@@H]1OC(O[C@H]2[C@H](O)C(COC(=O)\C=C\C3=CC=C(O)C(O)=C3)O[C@@H](OCCC3=CC(O)=C(O)C=C3)[C@@H]2O)[C@H](O)[C@H](O)[C@H]1O
Licodione	$C_{15}H_{12}O_5$	271.0611	OC1=CC=C(C=C1)C(=O)CC(=O)C1=CC=C(O)C=C1O
Lingstroside	$C_{25}H_{32}O_{12}$	523.1821	[H][C@]1(CC(=O)OCCC2=CC=C(O)C=C2)\C(=C\C)[C@H](O[C@@H]2O[C@H](CO)[C@@H](O)[C@H](O)[C@H]2O)OC=C1C(=O)OC
Lingstroside aglycone	$C_{19}H_{22}O_7$	361.1291	COC(=O)C1=CO[C@@H](O)\C(=C\C)[C@@H]1CC(=O)OCCC1=CC=C(O)C=C1

**Compound**	**Molecular Formula**	**[M-H]⁻ *m/z* calculated**	**SMILES**
Lingstroside aglycone dialdehydic form	$C_{19}H_{22}O_7$	361.1291	[H]C1=CC(CCOC(=O)C[C@]([H])(C(C=O)C(=O)OC)C(=C/C)\C=O)=CC=C1O
Lingstroside aglycone monoaldehydic form	$C_{19}H_{22}O_7$	361.1291	[H]C1=CC(CCOC(=O)C[C@@]2([H])C(C=O)C(C)OC=C2C(=O)OC)=CC=C1O
Luteolin 3.7–o-diglucoside	$C_{27}H_{30}O_{16}$	609.1461	OC[C@@H]1O[C@@H](OC2=CC(O)=C3C(=O)C=C(OC3=C2)C2=CC(O[C@@H]3O[C@@H](CO)[C@H](O)[C@@H](O)[C@@H]3O)=C(O)C=C2)[C@@H](O)[C@H](O)[C@H]1O
Luteolin-4-glucoside	$C_{21}H_{20}O_{11}$	447.0932	OC[C@@H]1OC(OC2=C(O)C=CC(=C2)C2=CC(=O)C3=C(O)C=C(O)C=C3O2)[C@@H](O)[C@H](O)[C@H]1O
Luteolin-7-o-glucoside	$C_{21}H_{20}O_{11}$	339.0932	OC[C@@H](O)[C@H]1O[C@@H](OC2=C(OC3=CC(O)=CC(O)=C3C2=O)C2=CC(O)=C(O)C=C2)[C@H](O)[C@H]1O
Maslinic acid	$C_{30}H_{48}O_4$	471.3484	CC1(C)CC[C@@]2(CC[C@]3(C)C(=CC[C@@H]4[C@@]5(C)C[C@@H](O)[C@H](O)C(C)(C)[C@@H]5CC[C@@]34C)[C@@H]2C1)C(O)=O
Methoxy quinone	$C_7H_6O_3$	137.0244	COC1=CC(=O)C=CC1=O
Methyl oleuropein aglycone	$C_{20}H_{24}O_8$	391.1412	CC\C=C1\[C@H](O)OC=C([C@H]1CC(=O)OCCC1=CC(O)=C(O)C=C1)C(=O)OC
Naringenin	$C_{15}H_{12}O_5$	271.0611	c1cc(ccc1[C@@H]2CC(=O)c3c(cc(cc3O2)O)O)O
Neo-nuzhenide	$C_{31}H_{42}O_{18}$	701.2298	COC(=O)C1=CO[C@@H](O[C@@H]2O[C@H](CO)[C@@H](O)[C@H](O)[C@H]2O)\C(=C/C)C1CC(=O)OC[C@@H]1O[C@H](OCCC2=CC(O)=C(O)C=C2)[C@@H](O)[C@H](O)[C@H]1O
Nuzhenide	$C_{31}H_{42}O_{17}$	685.2349	COC(=O)C1=CO[C@@H](O[C@@H]2O[C@H](CO)[C@@H](O)[C@H](O)[C@H]2O)\C(=C/C)C1CC(=O)OC[C@@H]1O[C@H](OCCC2=CC=C(O)C=C2)[C@@H](O)[C@H](O)[C@H]1O
Oleanolic acid	$C_{30}H_{48}O_3$	455.3535	CC1(C)CC[C@@]2(CC[C@]3(C)C(=CC[C@@H]4[C@@]5(C)CC[C@H](O)C(C)(C)[C@@H]5CC[C@@]34C)[C@@H]2C1)C(O)=O

**Compound**	**Molecular Formula**	**[M-H]$^-$ *m/z* calculated**	**SMILES**
Oleokoronal	$C_{19}H_{22}O_7$	361.1292	[[H]O\C([H])=C(/C(=O)OC)C([H])(CC(=O)OCCC1=CC=C(O)C([H])=C1)C(=C/C)\C=O
Oleomissional	$C_{19}H_{22}O_8$	377.1241	[H]O\C([H])=C(/C(=O)OC)C([H])(CC(=O)OCCC1=CC=C(O)C(O)=C1)C(=C/C)\C=O
Oleuropein aglycone	$C_{19}H_{22}O_8$	377.1241	[H][C@]1(CC(=O)OCCC2=CC=C(O)C(O)=C2)\C(=C/C)[C@H](O)OC=C1C(=O)OC
Oleuropein aglycone dialdehydic form	$C_{19}H_{22}O_8$	377.1241	[H][C@@](CC(=O)OCCC1=CC=C(O)C(O)=C1)(C(C=O)C(=O)OC)C(=C/C)\C=O
Oleuropein aglycone monoaldehydic form	$C_{19}H_{22}O_8$	377.1241	[H][C@]1(CC(=O)OCCC2=CC=C(O)C(O)=C2)C(C=O)C(C)OC=C1C(=O)OC
Olivil	$C_{20}H_{24}O_7$	375.1449	COC1=C(O)C=CC(C[C@@]2(O)CO[C@@H]([C@H]2CO)C2=CC(OC)=C(O)C=C2)=C1
Olivin	$C_{17}H_{16}O_6$	315.0874	COC1=C(O)C=CC(\C=C(/C)C(=O)C2=C(O)C=C(O)C=C2O)=C1
p- Hydroxybenzoic acid	$C_7H_6O_3$	137.0244	OC(=O)C1=CC=C(O)C=C1
Protocatechuic acid	$C_7H_6O_4$	153.0193	OC(=O)C1=CC(O)=C(O)C=C1
Quercetin-3-o-glucoside	$C_{21}H_{20}O_{12}$	355.0882	OC[C@@H](O)[C@H]1O[C@@H](OC2=C(OC3=CC(O)=CC(O)=C3C2=O)C2=CC(O)=C(O)C=C2)[C@H](O)[C@H]1O
Quercetin-3-rhamnoside	$C_{21}H_{20}O_{11}$	447.0932	C[C@@H]1O[C@@H](OC2=C(OC3=CC(O)=CC(O)=C3C2=O)C2=CC=C(O)C(O)=C2)[C@H](O)[C@H](O)[C@H]1O
Rutin	$C_{27}H_{30}O_{16}$	609.1461	CC1OC(OCC2OC(OC3=C(OC4=CC(O)=CC(O)=C4C3=O)C3=CC=C(O)C(O)=C3)C(O)C(O)C2O)C(O)C(O)C1O
Salidroside	$C_{14}H_{20}O_7$	299.1136	OCC1OC(OCCC2=CC=C(O)C=C2)C(O)C(O)C1O
Scopoletin	$C_{10}H_8O_4$	191.0349	COC1=C(O)C=C2OC(=O)C=CC2=C1
Secologanoside	$C_{16}H_{22}O_{11}$	389.1089	OC[C@H]1O[C@@H](O[C@@H]2OC=C([C@@H](CC(O)=O)[C@H]2C=C)C(O)=O)[C@H](O)[C@@H](O)[C@@H]1O

**Compound**	**Molecular Formula**	**[M-H]$^-$ *m/z* calculated**	**SMILES**
S-Hellicoside	$C_{29}H_{36}O_{17}$	655.1879	c1cc(c(cc1/C=C/C(=O)O[C@@H]2[C@H](O[C@H]([C@@H]([C@H]2O[C@H]3[C@@H]([C@H]([C@@H]([C@H](O3)CO)O)O)O)O)OC[C@H](c4ccc(c(c4)O)O)O)CO)O)O
Sinapic acid	$C_{11}H_{12}O_5$	223.0611	COC1=CC(\C=C\C(O)=O)=CC(OC)=C1O
Suspensaside	$C_{29}H_{36}O_{16}$	639.1930	C[C@H]1[C@@H]([C@H]([C@H]([C@@H](O1)OC[C@@H]2[C@H]([C@@H]([C@H]([C@@H](O2)OCC(c3ccc(c(c3)O)O)O)O)O)OC(=O)/C=C/c4ccc(c(c4)O)O)O)O)O
Taxifolin	$C_{15}H_{12}O_7$	303.0510	OC1C(OC2=CC(O)=CC(O)=C2C1=O)C1=CC=C(O)C(O)=C1
Tyrosol acetate	$C_{10}H_{12}O_3$	179.0713	CC(=O)OCCC1=CC=C(O)C=C1
Tyrosol glucoside	$C_{14}H_{20}O_7$	299.1136	[H][C@]1(CO)O[C@@]([H])(OCCC2=CC=C(O)C=C2)[C@]([H])(O)[C@@]([H])(O)[C@]1([H])O
Vanillic acid	$C_8H_8O_4$	167.0349	COC1=CC(=CC=C1O)C(O)=O
Verbascoside	$C_{29}H_{36}O_{15}$	623.1981	CC1OC(OC2C(O)C(OCCC3=CC=C(O)C(O)=C3)OC(CO)C2OC(=O)C=CC2=CC=C(O)C(O)=C2)C(O)C(O)C1O
Verucosin	$C_{20}H_{24}O_5$	343.1550	Oc1ccc(cc1OC)[C@@H]3O[C@H](c2cc(OC)c(O)cc2)[C@H]([C@@H]3C)C
Xanthonic acid	$C_{14}H_8O_4$	239.0349	OC(=O)C1=C2C(OC3=CC=CC=C3C2=O)=CC=C1

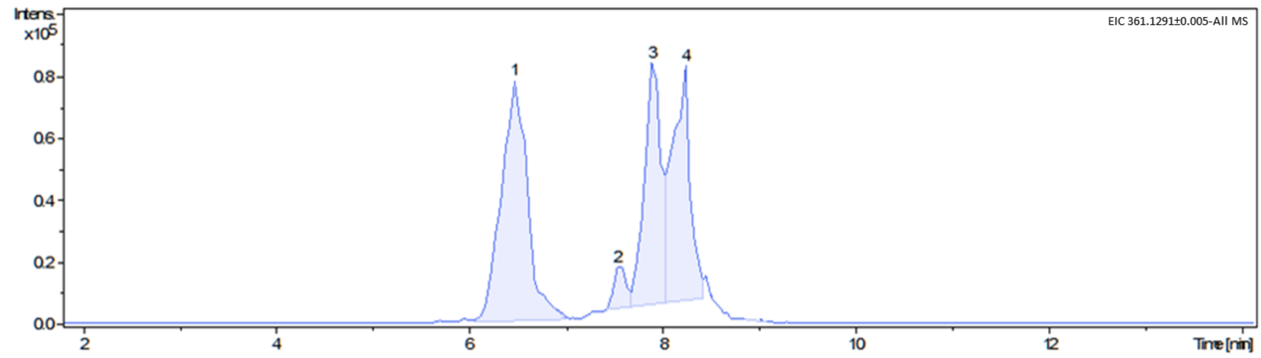

Figure S1. EIC of lingstroside transformation to: lingstroside aglycone (1); lingstroside aglycone monoaldehydic form (2); lingstroside aglycone dialdehydic form (3); oleokoronal (4).

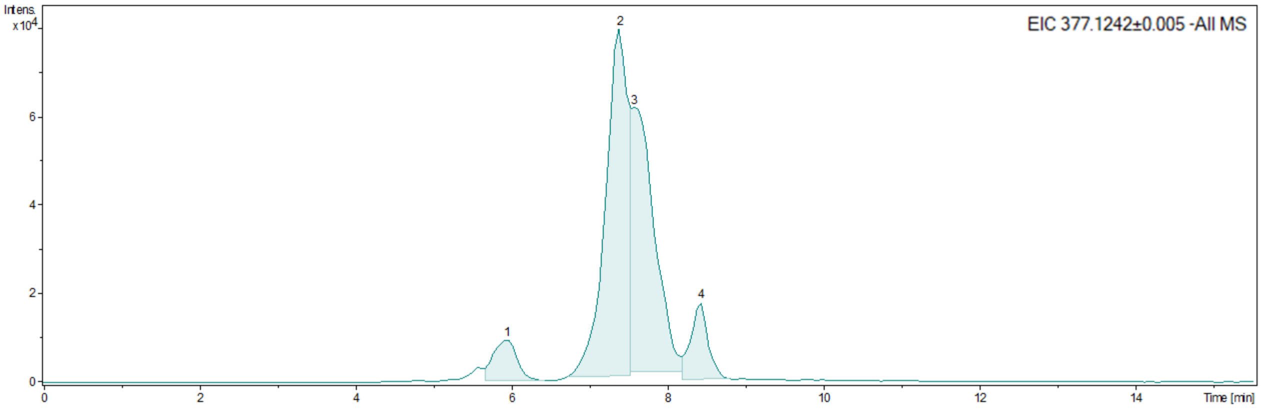

Figure S2. EIC of oleuropein transformation to: oleuropein aglycone (1); oleuropein aglycone monoaldehydic form (2); oleuropein aglycone dialdehydic form (3); oleomissional (4).

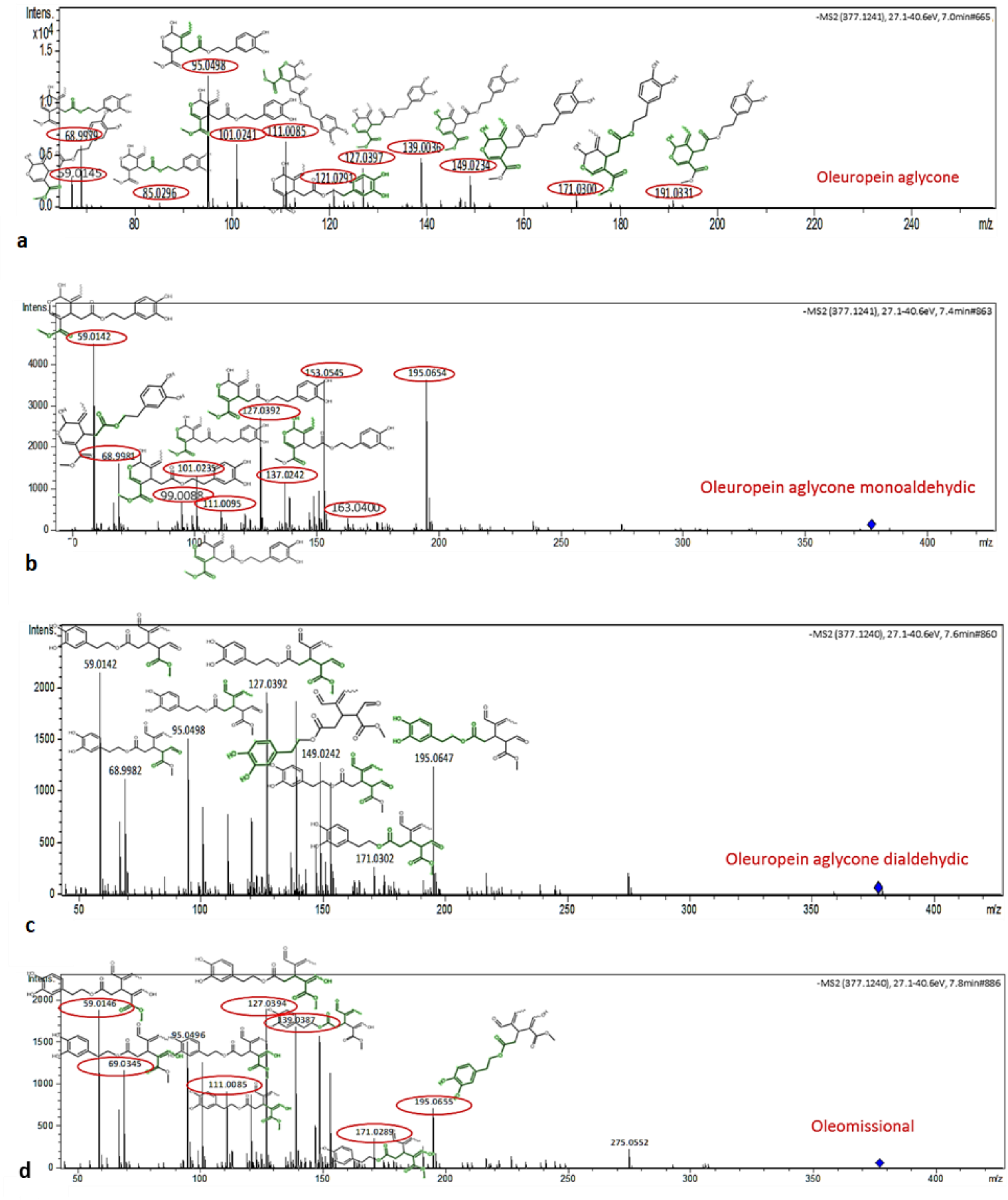

Figure S3. Characteristic spectra of: (**a**) oleuropein aglycone; (**b**) oleuropein aglycone monoaldehydic form; (**c**) oleuropein aglycone dialdehydic; (**d**) oleomissional.

Table S4. Suspect screening quantification results (mg/kg).

Sample	Hydroxytyrosol acetate (mg/kg)	Decarboxymethyl ligstroside aglycone (mg/kg)	Decarboxymethyl oleuropein aglycone (mg/kg)	10-Hydroxy-10-methyl oleuropein aglycone (mg/kg)	10-Hydroxy-decarboxymethyl oleuropein aglycone (mg/kg)	10-Hydroxy oleuropein aglycone (mg/kg)	Ligstroside aglycone (mg/kg)	Methyl oleuropein aglycone (mg/kg)	Oleuropein aglycone (mg/kg)	1-Acetoxypinoresinol (mg/kg)	1-Hydroxypinoresinol (mg/kg)	Syringaresinol (mg/kg)	Elenolic acid (mg/kg)	Hydroxylated from of elenolic acid (mg/kg)
ME1	4.02	5.50	6.34	15.1	2.52	7.78	291	11.4	360	3.45	1.40	0.36	2.32	nd
MO1	2.66	4.18	2.98	10.7	2.54	9.30	108	10.5	137	1.13	1.49	0.40	2.32	nd
MO3	3.04	4.76	3.84	16.2	2.76	7.38	162	8.70	192	1.70	1.73	0.35	2.34	nd
PA1	4.70	4.94	8.40	7.85	2.46	7.03	131	9.50	274	2.52	1.60	0.39	2.36	nd
PL1	3.92	4.72	6.80	7.98	2.88	6.61	66.2	7.92	165	2.68	1.73	0.37	2.32	nd
PA2	3.40	5.42	5.36	8.27	2.50	6.89	116	10.9	161	2.18	1.90	0.37	2.30	nd
AG1	3.20	3.86	4.24	24.4	2.52	6.70	120	10.4	229	2.06	1.15	0.37	2.30	nd
GE1	8.26	6.12	5.02	11.0	2.72	27.4	114	17.7	196	7.89	0.73	0.37	4.66	nd
ME2	7.50	4.56	3.22	14.5	2.44	33.3	8.24	13.7	127	2.81	0.68	0.40	2.30	nd
PR1	9.22	4.84	6.48	14.3	2.46	26.6	151	16.1	299	6.38	nd	0.37	7.16	nd
PA3	8.90	6.72	6.34	9.18	2.68	25.6	8.33	15.2	202	11.6	0.89	0.35	6.92	nd
PL3	5.84	5.82	5.16	7.27	nd	26.2	8.72	15.7	252	6.33	0.85	nd	2.38	nd
PH1	8.48	5.56	5.72	13.8	2.62	25.9	8.21	28.1	236	3.08	0.73	0.39	2.36	nd
AK1	3.68	5.62	5.70	31.4	2.72	9.80	159	7.81	236	1.90	1.22	0.41	2.38	nd
PH2	4.96	5.72	8.28	12.1	2.22	8.30	137	7.44	291	6.50	1.19	0.77	2.50	nd
PH3	5.28	5.54	8.80	15.6	2.18	8.02	140	8.46	335	5.50	1.10	0.63	2.42	nd
GE2	3.06	5.28	4.10	18.4	1.62	8.90	91.3	10.9	124	4.30	1.22	0.51	2.32	nd
GE3	4.58	4.90	7.52	20.7	2.02	7.80	131	7.96	274	4.30	1.10	0.53	2.38	nd
SK1	3.60	5.02	4.02	32.1	2.64	16.7	289	22.6	248	4.80	0.95	0.52	2.42	nd
DF1	nd	4.52	2.94	7.32	2.36	7.56	14.2	67.0	7.18	2.01	1.27	103	2.32	nd
GE4TH	2.30	4.12	3.88	8.69	2.38	8.17	16.2	113	6.49	3.06	1.09	124	2.32	nd
KO1	2.32	3.70	3.10	11.9	2.34	9.62	16.7	108	11	3.28	1.40	142	2.32	nd
KO3	2.34	3.66	3.94	12.4	2.38	11.0	20.5	85.3	14.5	2.13	1.21	134	2.32	nd
KO4	nd	4.12	5.96	11.6	2.46	7.53	20.7	52.1	8.11	2.15	1.18	158	2.32	nd

Sample	Hydroxytyrosol acetate (mg/kg)	Decarboxymethyl ligstroside aglycone (mg/kg)	Decarboxymethyl oleuropein aglycone (mg/kg)	10-Hydroxy-10-methyl oleuropein aglycone (mg/kg)	10-Hydroxy-decarboxymethyl oleuropein aglycone (mg/kg)	10-Hydroxy oleuropein aglycone (mg/kg)	Ligstroside aglycone (mg/kg)	Methyl oleuropein aglycone (mg/kg)	Oleuropein aglycone (mg/kg)	1-Acetoxypinoresinol (mg/kg)	1-Hydroxypinoresinol (mg/kg)	Syringaresinol (mg/kg)	Elenolic acid (mg/kg)	Hydroxylated from of elenolic acid (mg/kg)
KP1	nd	5.70	6.40	8.37	2.48	7.87	18.9	173	7.58	1.86	1.30	289	2.34	nd
GE4	nd	5.72	3.36	12.7	2.46	11.5	13.7	148	6.94	4.86	1.51	136	2.32	nd
GE5	nd	7.42	9.52	10.8	2.70	7.79	27	105	8.10	7.57	1.61	194	2.34	nd
GE6	nd	5.26	8.26	8.45	2.48	8.04	22.4	165	7.70	3.21	1.24	269	2.34	nd
KA1	2.30	4.92	6.90	8.32	2.44	7.98	21	80.7	8.74	1.55	1.23	160	2.42	nd
PL4	nd	5.20	5.04	6.93	2.40	nd	18.4	75.1	6.80	2.68	0.73	122	2.34	nd
M1	4.64	4.96	7.42	18.9	2.08	8.66	126	9.34	222	5.29	1.09	0.56	2.40	nd
M2	5.76	6.08	9.38	20.6	2.44	8.90	196	11.7	327	7.08	1.09	0.60	2.48	nd
M3	3.06	6.86	4.16	14.3	3.26	10.7	101	12.7	108	4.24	1.55	0.50	2.36	nd
M4	3.40	6.44	4.98	16.8	2.54	9.20	120	10.2	166	5.80	1.27	0.62	2.38	nd
EE1	4.12	6.50	4.98	22.9	2.44	10.8	210	13.7	191	6.09	1.23	0.88	2.42	nd
1087ELSG	4.48	11.2	11.2	2.82	0.37	0.29	69.5	2.02	108	20.3	nd	0.48	0.26	nd
1248ELOA	20.8	22.1	40.7	5.75	0.82	0.95	178	10.8	546	27.1	0.85	1.29	0.87	0.03
1298ELGP	22.5	21.0	47.2	3.20	nd	nd	114	10.3	266	14.3	2.22	1.16	0.21	nd
2039ELET	16.4	18.7	33.8	3.98	0.42	0.30	148	1.36	331	47.0	0.77	1.98	0.49	nd
2040LAZF	22.4	24.7	44.1	7.76	2.01	0.39	135	2.99	409	35.7	0.65	1.36	0.76	nd
2042LAIX	10.1	16.4	21.2	10.3	0.54	0.30	67.10	2.96	161	43.6	0.61	2.41	0.52	nd
2051LAPK	18.6	19.7	31.9	8.08	2.63	1.14	250	3.40	465	24.5	0.97	0.89	1.89	0.01
2052LATHG	7.78	9.78	13.7	6.95	0.26	0.73	49.0	4.79	116	13.8	0.72	1.12	0.13	0.02
2057LASI	9.04	12.7	21.7	1.36	0.48	nd	47.1	0.96	132	29.8	1.70	1.16	0.28	0.01
2085ELGG	15.8	14.1	30.2	2.98	0.52	0.13	57.3	2.02	188	12.9	0.19	0.46	0.16	nd
2093ELBB	12.9	13.1	29.1	2.27	0.33	0.11	47.9	1.61	160	24.5	nd	1.29	0.15	nd
2108ELEK	24.0	22.9	55.1	5.91	0.89	0.06	74.8	2.44	275	45.3	0.73	1.68	0.19	nd
2112ELNK	18.1	21.9	38.6	7.90	0.72	0.21	38.3	2.97	134	66.0	1.38	2.69	0.28	nd
2135LAEA	23.8	25.4	46.4	3.57	1.58	0.59	191	2.19	422	14.4	nd	0.67	2.01	nd
2136LAPK	28.9	31.9	53.7	20.2	3.99	2.37	293	16.7	732	24.4	0.55	1.23	1.97	0.02

Sample	Hydroxytyrosol acetate (mg/kg)	Decarboxymethyl ligstroside aglycone (mg/kg)	Decarboxymethyl oleuropein aglycone (mg/kg)	10-Hydroxy-10-methyl oleuropein aglycone (mg/kg)	10-Hydroxy-decarboxymethyl oleuropein aglycone (mg/kg)	10-Hydroxy oleuropein aglycone (mg/kg)	Ligstroside aglycone (mg/kg)	Methyl oleuropein aglycone (mg/kg)	Oleuropein aglycone (mg/kg)	1-Acetoxypinoresinol (mg/kg)	1-Hydroxypinoresinol (mg/kg)	Syringaresinol (mg/kg)	Elenolic acid (mg/kg)	Hydroxylated from of elenolic acid (mg/kg)
2137LAMS	11.2	19.7	23.1	92.2	0.32	1.96	75.2	19.6	185	12.1	nd	1.44	0.25	0.04
2138LATHM	14.2	21.1	27.4	4.35	0.47	0.29	75.2	1.67	178	39.7	nd	1.62	0.73	nd
2139LAOT	23.2	24.3	39.6	10.8	0.84	0.90	181	1.88	404	26.2	1.24	0.93	2.87	0.05
2140LANX	13.4	17.2	28.2	2.90	1.62	0.44	128	1.52	254	14.8	0.73	0.60	1.40	nd
2154ELME	15.8	15.2	28.9	3.53	1.63	0.64	104	7.55	299	30.7	1.32	1.16	0.43	nd
2155ELET	18.8	16.6	35.7	16.2	1.82	0.61	105	10.7	359	8.69	nd	0.72	0.24	nd
2169ELEK	18.4	18.4	41.3	3.29	1.48	0.17	86.7	6.57	294	10.8	0.58	nd	0.31	nd
2170ELGK	24.9	22.6	50.7	3.44	2.26	0.21	80.9	4.45	284	30.1	1.65	1.14	0.30	nd
2178LAOX	10.4	16.4	26.8	9.50	1.35	0.89	109	7.98	270	8.76	1.31	nd	0.45	nd
2179LAKZ	18.6	19.0	35.0	11.1	2.47	0.96	166	6.53	437	6.73	1.13	0.50	0.67	nd
2180LAGA	11.7	12.8	14.7	5.98	0.78	0.16	45.8	4.81	147	25.9	1.43	1.24	0.19	nd
2181LAKA	11.1	12.9	20.4	7.42	1.85	0.63	87.3	8.05	234	12.0	1.22	0.98	0.24	nd
2182LAID	9.11	16.5	17.0	5.77	1.76	0.31	99.4	6.63	183	1.27	1.01	nd	0.12	nd
2183LADK	11.4	15.5	25.1	7.70	1.24	0.34	62.9	6.07	168	19.1	0.87	0.86	0.18	nd
2225LAPT	6.72	10.2	8.02	7.82	0.11	1.93	61.4	2.06	175	28.0	1.06	1.19	0.52	0.15
2227ELNK	11.1	11.0	26.4	3.54	0.30	0.11	19.0	1.22	71.8	30.7	0.88	1.59	0.17	nd
2228ELEK	13.7	14.3	32.1	3.11	0.43	0.20	37.5	0.99	116	18.5	nd	1.04	0.16	nd
2238ELMK	15.6	12.4	38.6	4.94	1.39	0.18	47.9	2.68	175	7.53	1.10	nd	0.18	nd
2239ELND	6.07	6.18	12.5	1.08	0.09	0.17	23.2	2.16	80.4	10.9	nd	nd	0.07	nd
2242LAPK	11.5	12.4	27.9	8.11	1.64	0.76	121	4.20	324	22.1	0.71	1.03	0.75	nd
2243LAPK	9.60	12.5	24.0	4.22	0.99	0.67	133	6.73	338	66.5	nd	2.33	0.71	0.03
2251ELTHS	19.9	19.5	43.5	4.41	0.70	0.14	123	1.96	277	25.8	0.54	1.19	0.26	nd
2252ELXX	23.8	28.5	52.3	9.74	1.86	nd	79.9	1.31	243	65.1	0.86	1.94	0.27	nd
2278ELPA	8.44	7.43	17.5	9.92	0.96	2.20	208	8.53	460	21.4	1.17	0.96	0.70	nd
2290ELMK	22.3	18.4	46.4	15.5	2.30	0.52	66.2	6.71	328	42.2	1.80	1.69	0.26	0.02
2291ELLX	20.3	18.1	43.9	9.66	2.17	0.62	110	6.42	370	36.0	1.72	2.61	0.32	nd

Sample	Hydroxytyrosol acetate (mg/kg)	Decarboxymethyl ligstroside aglycone (mg/kg)	Decarboxymethyl oleuropein aglycone (mg/kg)	10-Hydroxy-10-methyl oleuropein aglycone (mg/kg)	10-Hydroxy-decarboxymethyl oleuropein aglycone (mg/kg)	10-Hydroxy oleuropein aglycone (mg/kg)	Ligstroside aglycone (mg/kg)	Methyl oleuropein aglycone (mg/kg)	Oleuropein aglycone (mg/kg)	1-Acetoxypinoresinol (mg/kg)	1-Hydroxypinoresinol (mg/kg)	Syringaresinol (mg/kg)	Elenolic acid (mg/kg)	Hydroxylated from of elenolic acid (mg/kg)
2293LAXG	16.1	15.5	32.6	55.9	0.93	2.35	150	18.6	447	84.4	nd	4.32	0.69	0.03
2309ELXX	5.11	8.75	8.50	3.97	0.06	0.22	37.6	2.28	109	59.2	1.35	2.36	0.09	0.02
2345LAMM	3.72	10.1	7.49	6.37	0.15	3.06	125	7.71	241	38.2	1.38	1.29	0.75	0.21
2346LANE	11.6	16.4	22.4	8.35	0.62	5.44	256	7.25	556	30.5	nd	2.66	3.87	0.57
2349ELGK	24.8	27.1	46.1	5.79	5.04	1.38	218	15.3	555	10.2	0.50	0.61	1.28	0.03
3014KAAK	7.52	15.8	22.4	1.72	0.43	nd	103	0.64	206	20.0	nd	0.80	0.26	nd
3095KANK	8.00	9.58	15.5	2.73	0.34	0.21	71.7	Nd	117	9.81	0.90	0.56	0.55	nd
3355KADL	10.8	17.5	24.3	1.55	0.93	1.92	223	9.73	486	24.5	1.17	0.95	3.18	0.07
4292PAGPS	12.1	12.7	19.6	38.6	0.25	2.00	112	10.7	281	9.90	2.87	0.89	0.49	0.11
4302PAKA	14.8	12.7	31.1	10.8	1.24	1.23	153	8.96	486	17.0	0.51	0.68	0.93	0.09
6356PAGG	6.51	3.27	0.89	0.68	nd	nd	28.0	6.02	82.0	10.6	0.60	1.52	0.17	0.07
7034PLXP	25.6	27.1	47.5	12.1	2.02	0.43	164	1.28	442	26.4	1.01	1.60	1.09	nd
7046MAED	17.8	27.2	39.5	15.1	2.43	1.24	232	4.33	518	29.0	0.60	1.25	1.61	0.06
7070MASK	7.84	12.3	17.1	2.47	0.64	0.18	88.3	1.05	174	9.81	nd	0.28	0.47	0.02
7097KADB	14.6	20.1	31.8	3.10	1.11	0.23	137	2.14	326	29.7	nd	1.14	0.30	nd
7099PLDF	5.14	7.50	10.9	5.38	0.09	0.16	17.5	3.06	63.1	24.9	1.02	1.33	0.16	0.01
7109PLDP	16.6	18.0	35.7	5.47	0.45	0.17	74.6	0.86	244	70.9	1.06	2.24	0.10	nd
7144PLAB	6.48	11.9	11.6	5.87	0.14	0.82	51.5	1.94	108	20.5	nd	1.01	0.62	nd
7153MASK	10.2	15.5	17.7	5.89	0.46	0.37	67.5	2.04	148	15.6	0.33	0.42	0.45	0.02
7198MANM	8.18	6.98	13.5	9.20	0.19	0.89	114	7.42	307	7.67	1.16	nd	0.88	nd
7280KALN	8.42	9.52	14.1	45.1	0.79	5.38	360	16.6	680	24.1	1.76	0.90	0.48	nd

nd: not detected

Table S5. Geographical region, altitude, type of farming and harvesting period of the *Kolovi* EVOOs.

Sample	Region	Altitude	Type of farming	Harvesting
ME1	Megalochori	High	Conventional	2016 – 2017
MO1	Moria	Low	Organic	2016 – 2017
MO3	Moria	Low	Organic	2016 – 2017
PA1	Palaiokipos	Medium	Conventional	2016 – 2017
PL1	Palaiochori	High	Organic	2016 – 2017
PA2	Palaiokipos	Medium	Conventional	2016 – 2017
AG1	Skopelos	Medium	Organic	2016 – 2017
GE1	Gera	Medium	Organic	2016 – 2017
ME2	Megalochori	High	Conventional	2016 – 2017
PR1	Parakila	Low	Conventional	2016 – 2017
PA3	Gera	Medium	Conventional	2016 – 2017
PL3	Palaiochori	High	Conventional	2016 – 2017
PH1	Pigi	Medium	Conventional	2016 – 2017
AK1	Akrasi	High	Conventional	2016 – 2017
PH2	Pigi	Medium	Conventional	2016 – 2017
PH3	Pigi	Medium	Organic	2016 – 2017
GE2	Gera	Medium	Conventional	2016 – 2017
GE3	Gera	Medium	Conventional	2016 – 2017
SK1	Aleuropodas	Medium	Conventional	2016 – 2017
DF1	Loutra	Low	Conventional	2016 – 2017
GE4TH	Gera	Medium	Conventional	2016 – 2017
KO1	Moria	Low	Conventional	2016 – 2017
KO3	Moria	Low	Conventional	2016 – 2017
KO4	Moria	Low	Conventional	2016 – 2017
KP1	Palaiochori	High	Conventional	2016 – 2017
GE4	Gera	Medium	Conventional	2016 – 2017
GE5	Gera	Medium	Conventional	2016 – 2017
GE6	Gera	Medium	Conventional	2016 – 2017
KA1	Kalloni	Low	Conventional	2016 – 2017
PL4	Palaiochori	High	Organic	2016 – 2017
M1	Gera	Medium	Organic	2016 – 2017
M2	Gera	Medium	Organic	2016 – 2017
M3	Tsilia	Low	Organic	2016 – 2017
M4	Tsilia	Low	Organic	2016 – 2017
EE1	Skopelos	Medium	Organic	2016 – 2017
1087ELSG	Keramia	Low	Conventional	2017 – 2018
1248ELOA	Asomatos	Medium	Conventional	2017 – 2018
1298ELGP	Keramia	High	Organic	2017 – 2018
2039ELET	Palaiokipos	Medium	Conventional	2017 – 2018
2040LAZF	Skopelos	High	Organic	2017 – 2018
2042LAIX	Plakados	Low	Conventional	2017 – 2018
2051LAPK	Skopelos	Medium	Conventional	2017 – 2018
2052LATHG	Skopelos	High	Organic	2017 – 2018
2057LASI	Kato Tritos	High	Conventional	2017 – 2018
2085ELGG	Skopelos	Medium	Organic	2017 – 2018
2093ELBB	Palaiokipos	Low	Conventional	2017 – 2018
2108ELEK	Mesagros	High	Conventional	2017 – 2018

Sample	Region	Altitude	Type of farming	Harvesting
2112ELNK	Plakados	Low	Conventional	2017 – 2018
2135LAEA	Gera	High	Organic	2017 – 2018
2136LAPK	Gera	High	Organic	2017 – 2018
2137LAMS	Gera	Medium	Conventional	2017 – 2018
2138LATHM	Gera	Low	Organic	2017 – 2018
2139LAOT	Gera	Low	Organic	2017 – 2018
2140LANX	Gera	High	Organic	2017 – 2018
2154ELME	Palaiokipos	Medium	Conventional	2017 – 2018
2155ELET	Palaiokipos	Medium	Conventional	2017 – 2018
2169ELEK	Palaiokipos	Medium	Conventional	2017 – 2018
2170ELGK	Mesagros	Medium	Conventional	2017 – 2018
2178LAOX	Gera	Medium	Conventional	2017 – 2018
2179LAKZ	Gera	Medium	Conventional	2017 – 2018
2180LAGA	Skopelos	-	Conventional	2017 – 2018
2181LAKA	Gera	Medium	-	2017 – 2018
2182LAID	Tarti	Low	Organic	2017 – 2018
2183LADK	Gera	Low	Organic	2017 – 2018
2225LAPT	Palaiokipos	High	Conventional	2017 – 2018
2227ELNK	Papados	Low	Conventional	2017 – 2018
2228ELEK	Palaiokipos	Medium	Conventional	2017 – 2018
2238ELMK	Mesagros	High	Conventional	2017 – 2018
2239ELND	Plakados	High	Conventional	2017 – 2018
2242LAPK	Mesagros	High	Conventional	2017 – 2018
2243LAPK	Palaiokipos	High	Conventional	2017 – 2018
2251ELTHS	Palaiokipos	Medium	Conventional	2017 – 2018
2252ELXX	Palaiokipos	Low	Conventional	2017 – 2018
2278ELPA	Mesagros	High	Conventional	2017 – 2018
2290ELMK	Palaiokipos	Medium	Conventional	2017 – 2018
2291ELLX	Mesagros	Medium	Conventional	2017 – 2018
2293LAXG	Palaiokipos	High	Conventional	2017 – 2018
2309ELXX	Palaiokipos	Medium	Conventional	2017 – 2018
2345LAMM	Plakados	Low	Conventional	2017 – 2018
2346LANE	Kato Tritos	High	Conventional	2017 – 2018
2349ELGK	Mesagros	Medium	Conventional	2017 – 2018
3014KAAK	Plomari	High	Conventional	2017 – 2018
3095KANK	Plomari	High	Organic	2017 – 2018
3355KADL	Plomari	High	Conventional	2017 – 2018
4292PAGPS	Polixnitos	High	Organic	2017 – 2018
4302PAKA	Vasilika	Medium	Organic	2017 – 2018
6356PAGG	Parakila	Medium	Conventional	2017 – 2018
7034PLXP	Pigi	Medium	Conventional	2017 – 2018
7046MAED	Afalonas	Medium	Conventional	2017 – 2018
7070MASK	Moria	Medium	Organic	2017 – 2018
7097KADB	Agiasos	High	Organic	2017 – 2018
7099PLDF	Loutra	Low	Organic	2017 – 2018
7109PLDP	Ippeio	Low	Organic	2017 – 2018
7144PLAB	Lampou Mili	Medium	Organic	2017 – 2018
7153MASK	Moria	Medium	Organic	2017 – 2018
7198MANM	Evergetoulas	Medium	Conventional	2017 – 2018

Sample	Region	Altitude	Type of farming	Harvesting
7280KALN	Agia Marina	Medium	Conventional	2017 – 2018

Table S6. Quality Control Results.

Compound	%RSD of Peak Area $n = 11$	%RSD of t_R (min) $n = 11$	Δm (±error, mDa), $n = 11$
Apigenin	2.57	0.05	-0.09
Gallic acid	2.79	0.08	0.10
Hydroxytyrosol	3.94	0.08	0.13
Oleuropein	3.18	0.06	-0.16
Tyrosol	4.46	0.04	-0.12

Table S7. Target list.

Compound	Molecular Formula	$[M-H]^-$ m/z Theoretical	t_R Standard (min)
Caffeic acid	$C_9H_8O_4$	179.0349	1.53
Ferulic acid	$C_{10}H_{10}O_4$	193.0506	1.40
Gallic acid	$C_7H_6O_5$	169.0142	1.25
Homovanillic acid	$C_9H_{10}O_4$	181.0506	1.50
p-Coumaric acid	$C_9H_8O_3$	163.0400	1.34
Syringic acid	$C_9H_{10}O_5$	197.0455	1.44
Hydroxytyrosol	$C_8H_{10}O_3$	153.0557	3.53
Tyrosol	$C_8H_{10}O_2$	137.0608	4.07
Vanillin	$C_8H_8O_3$	151.0400	4.73
Apigenin	$C_{15}H_{10}O_5$	269.0455	8.24
Epicatechin	$C_{15}H_{14}O_6$	289.0716	4.37
Luteolin	$C_{15}H_{10}O_6$	285.0404	7.55
Oleuropein	$C_{25}H_{32}O_{13}$	539.1770	5.96
Pinoresinol	$C_{20}H_{22}O_6$	357.1343	6.49
